# scBGEDA: deep single-cell clustering analysis via a dual denoising autoencoder with bipartite graph ensemble clustering

**DOI:** 10.1093/bioinformatics/btad075

**Published:** 2023-02-03

**Authors:** Yunhe Wang, Zhuohan Yu, Shaochuan Li, Chuang Bian, Yanchun Liang, Ka-Chun Wong, Xiangtao Li

**Affiliations:** School of Artificial Intelligence, Hebei University of Technology, Tianjin, China; School of Artificial Intelligence, Jilin University, Jilin, China; School of Artificial Intelligence, Jilin University, Jilin, China; School of Artificial Intelligence, Jilin University, Jilin, China; Zhuhai Laboratory of Key Laboratory of Symbol Computation and Knowledge Engineering of Ministry of Education, Zhuhai College of Science and Technology, Zhuhai, China; Department of Computer Science, City University of Hong Kong, Kowloon Tong, Hong Kong SAR; School of Artificial Intelligence, Jilin University, Jilin, China

## Abstract

**Motivation:**

Single-cell RNA sequencing (scRNA-seq) is an increasingly popular technique for transcriptomic analysis of gene expression at the single-cell level. Cell-type clustering is the first crucial task in the analysis of scRNA-seq data that facilitates accurate identification of cell types and the study of the characteristics of their transcripts. Recently, several computational models based on a deep autoencoder and the ensemble clustering have been developed to analyze scRNA-seq data. However, current deep autoencoders are not sufficient to learn the latent representations of scRNA-seq data, and obtaining consensus partitions from these feature representations remains under-explored.

**Results:**

To address this challenge, we propose a single-cell deep clustering model via a dual denoising autoencoder with bipartite graph ensemble clustering called scBGEDA, to identify specific cell populations in single-cell transcriptome profiles. First, a single-cell dual denoising autoencoder network is proposed to project the data into a compressed low-dimensional space and that can learn feature representation via explicit modeling of synergistic optimization of the zero-inflated negative binomial reconstruction loss and denoising reconstruction loss. Then, a bipartite graph ensemble clustering algorithm is designed to exploit the relationships between cells and the learned latent embedded space by means of a graph-based consensus function. Multiple comparison experiments were conducted on 20 scRNA-seq datasets from different sequencing platforms using a variety of clustering metrics. The experimental results indicated that scBGEDA outperforms other state-of-the-art methods on these datasets, and also demonstrated its scalability to large-scale scRNA-seq datasets. Moreover, scBGEDA was able to identify cell-type specific marker genes and provide functional genomic analysis by quantifying the influence of genes on cell clusters, bringing new insights into identifying cell types and characterizing the scRNA-seq data from different perspectives.

**Availability and implementation:**

The source code of scBGEDA is available at https://github.com/wangyh082/scBGEDA. The software and the supporting data can be downloaded from https://figshare.com/articles/software/scBGEDA/19657911.

**Supplementary information:**

Supplementary data are available at *Bioinformatics* online.

## 1 Introduction

The cell is the basic unit of growth and development of an organism and has unique biological functions. The heterogeneity between cells in a cell population has isogenic properties, which can ascend from stochastic expression of genes, proteins and metabolites (Syed *et al.*, 2019). Conventional bulk RNA sequencing (RNA-seq) averages the transcriptional profiles of cells in a population, ignoring cell–cell heterogeneity in transcription (Ben-Dor *et al.*, 1999). The recent advances in single-cell RNA sequencing (scRNA-seq) technology allow measuring transcriptomes and understanding disease dysregulation at the single-cell resolution (Aviv *et al.*, 2017; Zhuohan *et al.*, 2023). However, scRNA-seq transcript expression profiles are particularly sparse due to low RNA capture rates, leading to spurious zero-count observations (Angerer *et al.*, 2017). Moreover, scRNA-seq data have high dimensionality and massive noise and often have very non-linear complex structures, which pose a major challenge for designing effective computational models.

Annotation of cell types by unsupervised learning, called clustering, is one of the first and most important steps of scRNA-seq data analysis; however, these constraints of the original scRNA-seq data make the process tricky. Autoencoder is a deep neural network that learns data representation using an encoder and a decoder in an unsupervised way. It is worth noting that the autoencoder realizes non-linear dimensionality reduction by projecting high-dimensional data into a low dimension in the latent space and then reconstructing the denoised data at the same time. Recently, a succession of deep embedded clustering (DEC) algorithms inspired by autoencoder were developed; for instance, Li *et al.* proposed DESC, which optimizes the objective function iteratively to achieve the clustering result and combines a deep autoencoder network with the clustering loss (Li *et al.*, 2020). Eraslan *et al.* developed a depth-counting autoencoder network named deep count autoencoder (DCA) to denoise the scRNA-seq data (Eraslan *et al.*, 2019). Further, Tian *et al.* designed single-cell model-based deep embedded clustering method (scDeepCluster) to cluster scRNA-seq data by combining DCA and DEC to conduct the dimension reduction and clustering process, respectively (Tian *et al.*, 2019). In particular, DCA and scDeepCluster apply a zero-inflated negative binomial (ZINB) model to capture the non-linear structure of scRNA-seq data. Chen *et al.* investigated a single-cell zero-inflated deep soft K-means (scziDesk) model to further exploit the clustering performance of ZINB using a soft *K-means* loss (Chen *et al.*, 2020). Most of those algorithms employ *K-means* clustering to generate the initial center points for KL loss to optimize the cluster results. However, it is hard to believe that such a single pattern can always perform well on all the scRNA-seq datasets.

Recently, emerging ensemble clustering methods have been demonstrated to naturally capture multiple scenarios to produce a consensus clustering result based on the consensus function; for instance, Kiselev *et al.* developed single-cell consensus clustering (SC3) algorithm to integrate basic clusterings into the final clustering solution by a hierarchical clustering (Kiselev *et al.*, 2017). Gan *et al.* proposed a consensus clustering framework using an ensemble strategy to fuze multiple basic clustering results (Gan *et al.*, 2018). Yang *et al.* proposed a SAFE-clustering method that combines solutions from four different methods with three hypergraph-based partitioning algorithms (Yang *et al.*, 2019). Huh *et al.* presented a SAME-clustering which uses clustering results from different methods and chooses a subset of maximum diversity to generate an ensemble solution (Huh *et al.*, 2020). Motivated by the above observations, ensemble clustering of compressed features obtained from deep autoencoders could be a good alternative for analyzing single-cell sequencing data, and even though there may be some cell types that are not necessarily completely precise, ensemble clustering methods tend to have advantages over each individual method.

In our study, we propose a deep single-cell clustering model via a dual denoising autoencoder with bipartite graph ensemble clustering, called scBGEDA, to perform clustering of scRNA-seq data. The scBGEDA pipeline consists of three core modules. The first module preprocesses the high-dimensional sparse scRNA-seq data into compressed low-dimensional data. The second module is a single-cell denoising autoencoder based on a dual reconstruction loss that characterizes the scRNA-seq data by learning the robust feature representations. In particular, by simultaneously optimizing the dual reconstruction loss and mean square error (MSE) loss, scBGEDA jointly improves the feature representation information of each cell preserved in an end-to-end manner. The third module comprises a bipartite graph ensemble clustering method used on the learned latent space to obtain the optimal clustering result. By developing a dual denosing autoencoder to capture the robust latent representations of scRNA-seq data, our scBGEDA algorithm encodes the scRNA-seq data in a discriminative representation, on which two decoders are trained to reconstruct the scRNA-seq data. Furthermore, bipartite graph ensemble clustering is proposed to address the clustering process, which is equivalent to solve the generalized eigen-problem to refine the clustering result. Multiple comparisons were conducted on 20 real scRNA-seq datasets from diverse sequencing platforms. The experimental results demonstrated the superior performance of the proposed algorithm, scBGEDA, compared with other clustering methods in several perspectives. We also carried out an extensive analysis on a large-scale scRNA-seq dataset to demonstrate that our algorithm is capable of dealing with large-scale data. Furthermore, functional gene analyses were carried out to further validate the effectiveness and interpretability of the scBGEDA model. The results indicated that scBGEDA may be adopted as a promising model for clustering scRNA-seq data.

## 2 Materials and methods

### 2.1 Methodology overview of scBGEDA

**Fig. 1.**
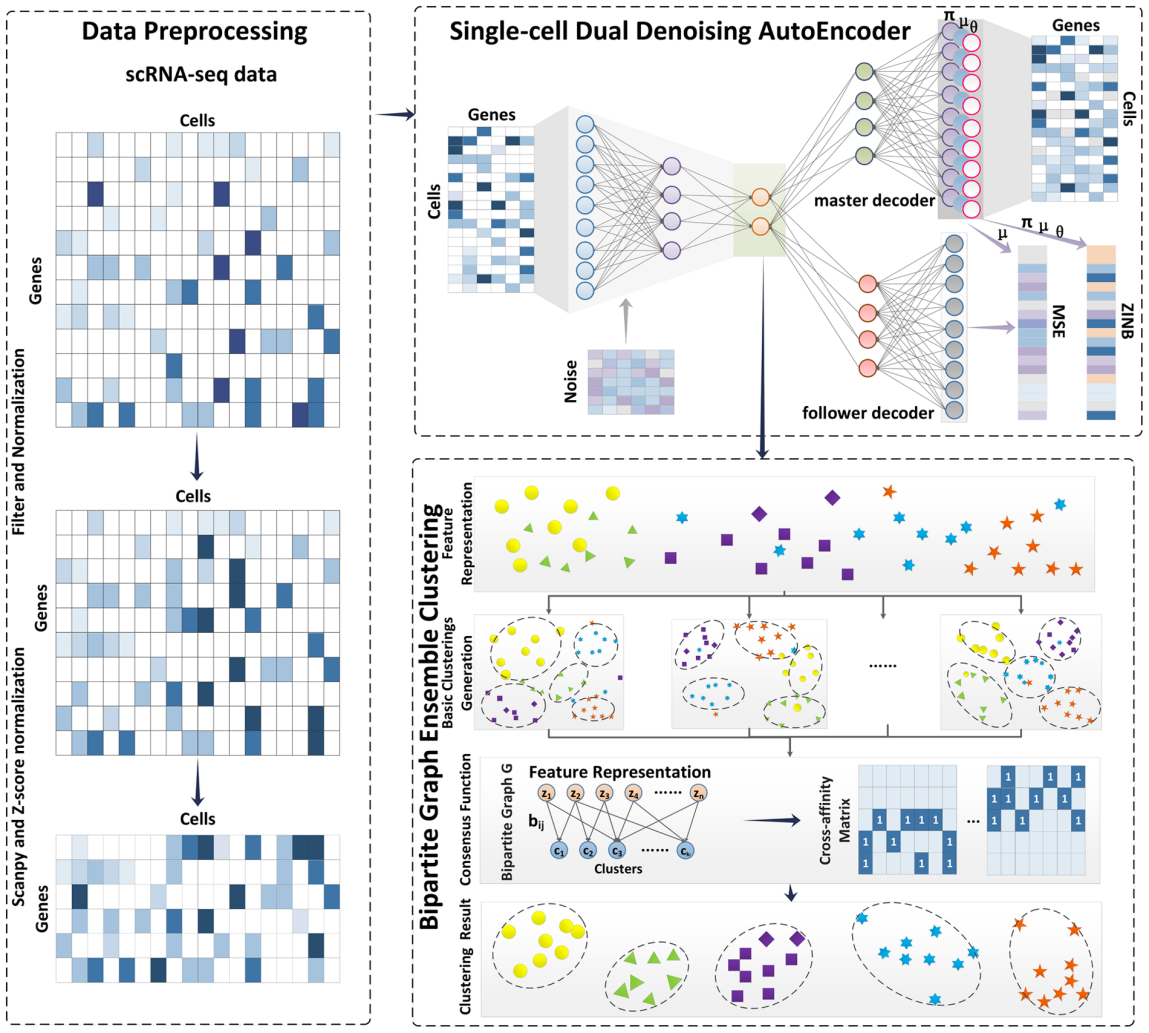
The overall workflow of the scBGEDA pipeline, comprising three components: the data preprocessing mechanism, the single-cell dual denoising autoencoder network and the bipartite graph ensemble clustering method

In our study, we propose scBGEDA for effective exploration of cell and gene representation in scRNA-seq data. The framework of scBGEDA has three components, including a data processing step to model the high-dimensional scRNA-seq data, a single-cell dual denoising autoencoder network and a bipartite graph ensemble clustering algorithm (Fig. 1). We propose a single-cell dual denoising autoencoder that incorporates the ZINB model into the denoising autoencoder network, to better capture the structure of the scRNA-seq data. The encoder of the second module intakes the preprocessed gene expression matrix after data filtering and normalization. The latent representation of the scRNA-seq data is reconstructed through the master decoder and follower decoder. Then, we design a bipartite graph ensemble clustering method in scBGEDA based on the bipartite graph and transfer cut approach inspired from ensemble clustering (Huang *et al.*, 2019). It comprises two phases, in the first, a set of basic clusterings are generated by the *K-means* clustering method; in the second, we produce a bipartite graph adopting both samples and clusters as the graph nodes to perform the consensus function by incorporating the multiple basic clusterings. Finally, the consensus clustering result is provided by solving the generalized eigen-problem.

### 2.2 Data collection and preprocessing

We collected 20 real scRNA-seq datasets from different species and organs available from various sequencing platforms (Drop-seq, 10x, inDrop, CEL-seq2 and Smart-seq2). Their characteristics are detailed in Supplementary Table S1, showing the source organ, the platform, the number of cell types, the number of cells, the zero percentage and the reference. Specifically, the Quake_10x_Trachea dataset is a single-cell transcriptome of the trachea, including 11 269 cells of 5 groups and a zero observation rate of 93.66%; the Tosches_turtle dataset contains 18 664 cells from the Drop-seq platform with 15 cell types and a zero observation rate of 90.83%; the Bach dataset from the 10x genomics platform has 23 184 cells, 8 cell types and a zero observation rate of 88.04%; and the Chen dataset is from Drop-seq with 12 089 cells, 46 cell types and a zero observation rate of 93.74%. In our study, these four scRNA-seq datasets were marked as large-scale datasets while the remaining sixteen scRNA-seq datasets have no more than 10 000 cells, and were marked as small-scale datasets.

ScBGEDA adopts the scRNA-seq gene expression matrix Y with *n* samples as the input. Since there is a large amount of technical and biological noise in the stochastic single-cell gene expression pattern, we first filter the genes that have almost no expression value. Then, we normalize the matrix by multiplying the division result between each row and each row’s sum by the total expression values’ medians of all cells, and transform them using the nature log scale in a continuous form. Afterwards, to further discard the genes having low identification and descriptive information, the top *m* highly variable genes are chosen by the filter_genes_dispersion function in single-cell analysis in python (Scanpy) package (Wolf *et al.*, 2018). Finally, the gene expression data are transferred by *Z*-score normalization with zero mean and unit variance. We record that normalized scRNA-seq matrix as an n×m scRNA-seq data $X=\{X_{1},X_{2},\ldots ,X_{i},\ldots ,X_{n}\},i=\{1,2,\ldots ,n\}$ and its corresponding original count matrix as $\bar{X}$ for data modeling.

### 2.3 Single-cell dual denoising autoencoder network

The single-cell dual denoising autoencoder network is based on the ZINB model to learn the latent feature representation of the scRNA-seq data. It captures the representation embedding of the scRNA-seq expression matrix by stacked layers in the encoder and decoder. The encoder is used to map the scRNA-seq data matrix X into the low-dimensional latent feature representation Z, extracting the unique information from the inputs. The dimension of Z is much smaller than that of X to avoid the ‘curse of dimensionality’ (Xie *et al.*, 2016). To prevent the overfitting phenomenon in deep learning, the input scRNA-seq data are corrupted with the random Gaussian noise, then, the autoencoder is constructed with fully connected layers. Therefore, the mapping function of encoder can be defined as follows:

(1) $$X^{\text{corrupt}}=X+e,\;Z=f^{W}\;\left(X^{\text{corrupt}}\right),$$

where X is the input scRNA-seq expression matrix, *e* is the random Gaussian noise that can be incorporated into each layer of the encoder, $X^{\text{corrupt}}$ is the corrupted data of the input, $f^{W}$ is the encoder function, *W* is the learnable weights of the function and Z is the output feature representation vector of the encoder.

The decoder takes the latent feature representation Z as the input, aiming to reconstruct the input from the low-dimensional feature representation Z. Due to the inevitable trade-off between reconstruction and clustering tasks, reconstruction loss is commonly the secondary optimum for clustering. Generally, the reconstruction loss is mainly determined by the distribution of the latent space and the reconstruction capacity of the decoder. However, the reconstruction capacity of the decoder network is unnecessary in the clustering procedure. To generate more discriminative features for the cluster assignments of scRNA-seq data, we construct a follower decoder to approximate the master decoder based on ZINB. The decoder of the dual denoising autoencoder network can be defined as follows:

(2) $${\hat{X}}_{1}=f^{W_{1}^{\prime }}\left(Z\right),\;{\hat{X}}_{2}=f^{W_{2}^{\prime }}\left(Z\right),$$

where $f^{W_{1}^{\prime }}$ and $f^{W_{2}^{\prime }}$ are the functions of the master decoder and follower decoder, $W_{1}^{\prime }$ and $W_{2}^{\prime }$ represent the weight parameter matrices and ${\hat{X}}_{1}$ and ${\hat{X}}_{2}$ are the reconstruction of inputs for the two decoders, respectively. To capture the characteristics of scRNA-seq data, the master decoder adopts ZINB autoencoder model-based loss to characterize the raw count data. Specifically, ZINB is used for mathematical modeling of dropout events in scRNA-seq data based on a combination of zero component and NB distribution, which can be defined as follows:

(3) $$\text{ZINB}\left(\bar{X}|\pi ,\mu ,\theta \right)=\pi \delta_{0}\left(\bar{X}\right)+\left(1-\pi \right)\times \text{NB}\left(\bar{X}|\mu ,\theta \right),$$

(4) $$\text{NB}\left(\bar{X}|\mu ,\theta \right)=\frac{\Gamma \left(\bar{X}+\theta \right)}{\Gamma \left(\bar{X}+1\right)\Gamma \left(\theta \right)}\times {\left(\frac{\theta }{\theta +\mu }\right)}^{\theta }\times {\left(\frac{\mu }{\theta +\mu }\right)}^{\bar{X}},$$

where $\bar{X}$ is the original raw count matrix and π is the probability of dropout events and μ and θ are the mean and dispersion in the negative binomial distribution, respectively, and are the parameters to be estimated. To model the ZINB distribution, the decoder network has three output layers to compute the three sets of parameters. The estimated parameters can be defined as follows:

(5) $$\begin{array}{c} {\hat{X}}_{1}=f^{W_{1}^{\prime }}\left(Z\right)\\ \Pi =\text{sigmoid}\;\left({\hat{X}}_{1}W_{\pi }\right)\\ M=\text{exp}\;\left({\hat{X}}_{1}W_{\mu }\right)\\ \Theta =\text{exp}\;\left({\hat{X}}_{1}W_{\theta }\right) \end{array},$$

where Π, *M* and Θ denote the matrix form of the estimations of π, μ and θ. Since the mean and dispersion parameters are non-negative values, we choose the exponential activation function for them. In terms of the additional coefficient π, the suitable activation function for it is sigmoid because the interval of π is between 0 and 1. The reconstruction loss function of the master decoder takes the negative log of ZINB likelihood, which can be expressed as follows:

(6) $$L_{1}(\pi ,\mu ,\theta |\bar{X})=-\text{log}\;\left(\text{ZINB}\;\left(\bar{X}|\pi ,\mu ,\theta \right)\right).$$

For the follower decoder, it is proposed to approximate the master decoder by transferring the latent representation Z to reconstruct the mean μ parameters in the ZINB model-based loss. In this manner, the follower decoder makes this dual denoising autoencoder model robust by exclusion rather than inclusion. Therefore, the loss of the follower decoder can be written as:

(7) $${\hat{X}}_{2}=f^{W_{2}^{\prime }}\left(Z\right),\;L_{2}\left(\mu ,{\hat{X}}_{2}\right)={\left\|\mu -{\hat{X}}_{2}\right\|}_{F}^{2},$$

where $\|\cdot {\|}_{F}$ is the Frobenius norm. It is the conventional MSE loss function and takes the ReLU function as the activation function.

To guarantee the quality of the feature representations in the latent space, the MSE loss is added to the original reconstruction loss, producing a dual reconstruction loss to learn the decoder network. The learning process of the dual denoising autoencoder aims to train the model by minimizing the dual objective loss function, which can be defined as follows:

(8) $$\begin{array}{c} L_{h}(\pi ,\mu ,\theta ,Z|\bar{X})=\lambda L_{1}+\gamma L_{2}\\ \min L_{h}(\pi ,\mu ,\theta ,Z|\bar{X})=\min\lambda (-\text{log}\;\left(\text{ZINB}\;\left(\bar{X}|\pi ,\mu ,\theta \right)\right))\\ +\gamma ({\left\|\mu -f^{W_{2}^{\prime }}\left(Z\right)\right\|}_{F}^{2}) \end{array},$$

where γ and λ are the hyperparameters to control the relative impact of $L_{1}$ and $L_{2}$.

### 2.4 Basic clustering generation

After obtaining the scRNA-seq data representation $Z=\left\{z_{1},z_{2},\ldots ,z_{n}\right\}$ with *n* samples from the latent space, our proposed model scBGEDA intends to exploit the relationship between the samples in Z and identify the cell types of scRNA-seq data by the bipartite graph ensemble clustering. At first, to ensure a fast running time for clustering the scRNA-seq datasets, we adopt the *K-means* clustering algorithm to produce a set of basic clusterings, which can be represented as follows:

(9) $$\Psi =\left\{\psi^{1},\psi^{2},\ldots ,\psi^{K}\right\},$$

where $\psi^{i}$ represents the *i*th basic clustering. We note that the number of clusters $k^{i}$ in $\psi^{i}$ is an integer randomly chosen from $k_{\max}$ and $k_{\min}$, where $k_{\min}$ and $k_{\max}$ represent the lower bound and upper bound of the cluster number, respectively.

### 2.5 Bipartite graph generation

To achieve a robust consensus clustering result, we adopt both samples and clusters as graph nodes Huang *et al.* (2019), a bipartite graph *G* can be defined as follows:

(10) G={Z,ϕ,B},

where Z is the feature representation; ϕ is the cluster set, and can expressed as follows:

(11) $$\phi =\left\{C^{1},C^{2},\ldots ,C^{k_{c}}\right\},\;k_{c}=\sum_{i=1}^{K}k_{i},$$

where $C^{i}$ is the *i*th cluster, $k_{i}$ is the number of clusters in the basic clustering $\psi^{i}$ and $k_{c}$ is the total number of clusters in Ψ. Moreover, *B* stands for the cross-affinity matrix that reflects the relationship between Z and ϕ, defined as follows:

(12) $$\begin{array}{c} B={\left\{b_{ij}\right\}}_{n\times k_{c}},\\ b_{ij}=\{\begin{array}{cc} 1, & \;\text{if}\;z_{i}\in C_{j},\\ 0, & \;\text{otherwise}.\; \end{array} \end{array}$$

It conveys that there is an edge between two nodes if, and only if, one node is a sample and the other is the cluster that contains that sample.

### 2.6 Bipartite graph ensemble clustering

After bipartite graph generation, we observe that it is equivalent to solve the generalized eigen-problem (Shi and Malik, 2000) in the spectral clustering, which can be denoted as:

(13) $$\begin{array}{c} Lu=\gamma \tau u\\ L=\tau -E\\ E=\left[\begin{array}{cc} 0 & B^{⊤}\\ B & 0 \end{array}\right] \end{array},$$

where *L* is the Laplacian matrix, $\tau \in R^{\left(n+k_{c})\times (n+k_{c}\right)}$ is the degree matrix and *E* is the full affinity matrix of *G*, $n+k_{c}$ is the number of nodes in *G*, since Z∪ϕ are the nodes in *G*.

However, taking *G* as a general graph is not computationally suitable for large-scale datasets. It has been demonstrated that solving the eigen-problem on graph *G* is equivalent to solve it on a much smaller graph (Li *et al.*, 2012). Therefore, to reduce the complexity to exploit the bipartite structure, we employ the transfer cut (Li *et al.*, 2012) to efficiently partition the graph *G* by transferring the eigen-problem with *G* to the eigen-problem with a smaller graph $G_{R}$ (with $k_{c}$ nodes). In particular, $G_{R}$ is conducted as $G_{R}=\left\{R,E_{R}\right\}$, which consists of the node set R and the affinity matrix $E_{R}=B^{⊤}{\tilde{\tau }}_{Z}^{-1}B$ (${\tilde{\tau }}_{Z}\in R^{n\times n}$ is a diagonal matrix). Then, the eigen-problem on $G_{R}$ can be formulated as follows:

(14) $$L_{R}v=\lambda \tau_{R}v,$$

where $L_{R}=\tau_{R}-E_{R}$ is the Laplacian for $G_{R}$ and $\tau_{R}\in R^{k_{c}\times k_{c}}$ is the degree matrix for $G_{R}$. According to the first *k* eigenvectors $\left\{v_{1},v_{2},\ldots ,v_{k}\right\}$ for $G_{R}$, the first *k* eigenvectors $\left\{u_{1},u_{2},\ldots ,u_{k}\right\}$ for *G* can be calculated (Huang *et al.*, 2019). Finally, the consensus clustering result is provided using *K-means* clustering on the new matrix through stacking $\left\{u_{1},u_{2},\ldots ,u_{k}\right\}$. We calculate the time complexity of scBGEDA in Supplementary Section S1.

## 3 Results

### 3.1 Model parameter settings

In our study, we trained each model, obtained the clustering results of the scRNA-seq data to evaluate the competitive methods. In scBGEDA, 2000 highly variable genes (m=2000) were picked as input of the single-cell dual denoising autoencoder network. The size of the hidden layers of the encoder network was 256 and 32. The setting of the decoder network was the opposite of that of the encoder. Hence, the size of the bottleneck layer was 32, indicating that the dimension of the latent representation was 32. During the training process, we adopted the Adam optimizer with a learning rate of 0.0001 to update the autoencoder and set the mini batch size to 256. Further, the default values of λ and γ were 1 and 0.00001 in the loss function of the model. Finally, the number of basic clusterings was fixed to 100 (K=100), and the upper and lower bounds of the number of clusters were set to 2 and 60 ($k_{\min}=2$, $k_{\max}=60$), respectively. The hyperparameter selection is discussed in Supplementary Sections S2–S4.

### 3.2 Related methods from the literature

Multiple existing computational methods were chosen for a comparative analysis of scRNA-seq data. First, we compared seven scRNA-seq data clustering algorithms to our proposed algorithm scBGEDA including hyper-fast with accurate processing via ensemble random projection (SHARP) (Wan *et al.*, 2020), clustering through imputation and dimensionality reduction (CIDR) (Lin *et al.*, 2017), semi-soft clustering with pure cells (SOUP) (Zhu *et al.*, 2019), spatial reconstruction model (Seurat) (Satija *et al.*, 2015), SC3 (Kiselev *et al.*, 2017), Scanpy (Wolf *et al.*, 2018) and principal components analysis (PCA) (Tian *et al.*, 2019). Then, we compared scBGEDA with four deep learning-based models including DCA (Eraslan *et al.*, 2019), scDeepCluster (Tian *et al.*, 2019), scziDesk (Chen *et al.*, 2020) and an unsupervised deep embedding algorithm (DESC) (Li *et al.*, 2020). The clustering evaluation metrics including NMI, ARI and two biological metrics (ASW and cLISI) are detailed in Supplementary Section S5. In addition, we have added the experiment to optimize the hyperparameters for those four deep learning-based competitors in a similar way to our study in Supplementary Section S6 and their hyperparameter optimizations are summarized in Supplementary Tables S3–S11.

### 3.3 Evaluations on real data

**Fig. 2.**
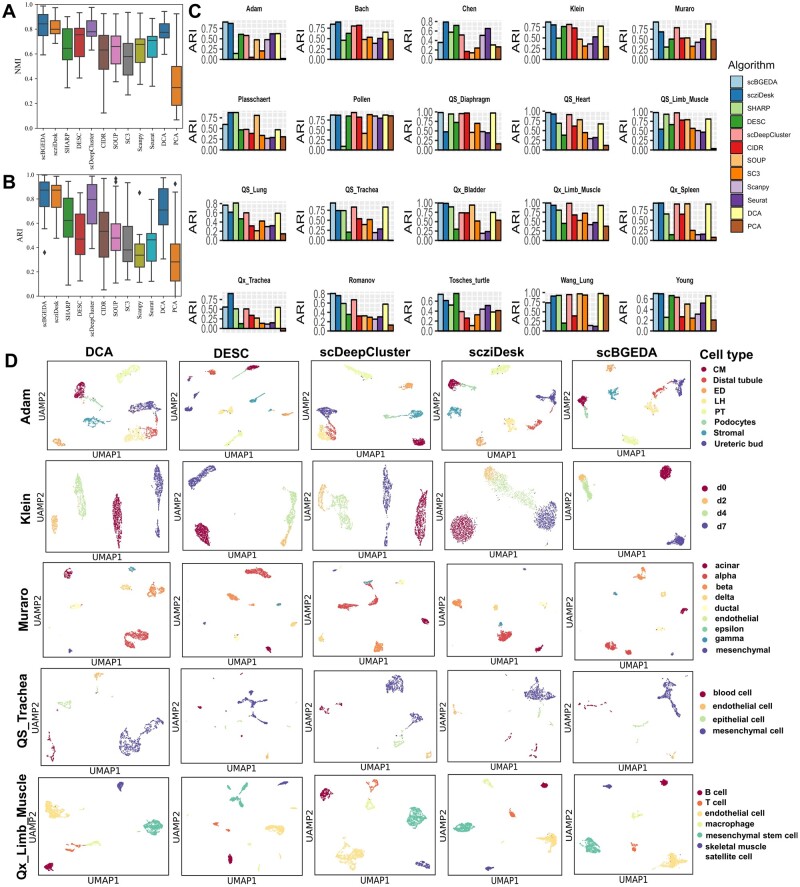
Real scRNA-seq data analysis results. (**A and B**) Box plots of ARI and NMI values on the 20 real scRNA-seq datasets with different clustering methods, respectively. The *X*-axis denotes the method and the *Y*-axis the ARI or NMI value. (**C**) Clustering performance comparison of the different clustering algorithms on the 20 real scRNA-seq datasets measured by ARI, the *X*-axis denotes the method and the *Y*-axis the ARI value. (**D**) 2D-visualization of the feature representations for five scRNA-seq datasets, Adam, Klein, Muraro, QS_Trachea and Qx_Limb_Muscle, learned by scBGEDA and four other deep learning-based algorithms. Each color in the cell-type panel on the outermost right side denotes a specific cell type

To demonstrate the effectiveness of scBGEDA, we used the 11 state-of-the-art clustering algorithms described above to compare to scBGEDA clustering on 20 real scRNA-seq datasets. To ensure the reliability of the clustering results for each method, we ran all methods 10 times under 10 random seeds, including 1111, 2222, … , 9999 and 10 000. After obtaining 10 ARI and NMI values, we computed average values to estimate the performance of each method. The experimental results are summarized in Figure 2A and B and Supplementary Tables S14–S17 measured by NMI, ARI, ASW and cLISI. As observed, scBGEDA provides the highest average NMI and ARI values of all the clustering methods. We also show a dot plot in Supplementary Figure S1A, where the scatter size represents the score rank of the methods and the color represents the ARI level score value. The results of the NMI comparison (Supplementary Fig. S1B) are almost identical to those of the ARI comparison. It can be observed that scBGEDA is orange or red with the biggest scatter in most datasets, always ranking in the top 3 of the 12 methods, elaborating the effectiveness of our proposed algorithm.

In Figure 2C and Supplementary Table S15, the ARI performance of each method on the 20 real datasets indicates that scBGEDA and scziDesk produce ARI values >0.6 on most scRNA-seq datasets. On Bach, Chen, Plasschaert, Qx_Spleen, Qx_Trachea and Wang_Lung datasets, scziDesk achieves better ARI results than scBGEDA. However, for the all other datasets, our proposed model scBGEDA is superior to scziDesk, with a 19% better ARI value on the large-scale scRNA-seq dataset QS_Trachea. DESC, scDeepCluster and DCA are surpassed by the other methods on only one scRNA-seq dataset, while SHARP performs best among all the methods on two scRNA-seq datasets. In addition, PCA has the lowest ARI value, even lower than 0.1, on 5 out of 20 scRNA-seq datasets. In terms of NMI values (Supplementary Fig. S1B and Supplementary Table S3), our proposed scBGEDA outperformed the other methods on 11 out of the 20 scRNA-seq datasets. Of note, compared to the other deep-learning models (scziDesk, DCA, DESC and scDeepCluster), scBGEDA obtains the best clustering performance on 11 out of the 20 datasets, demonstrating that scBGEDA obtains a more discriminative latent space. Moreover, to assess the variability of ARI and NMI values for significant differences, we calculate the Wilcoxon test to test the significant differences for those datasets. The Wilcoxon analysis results on those 20 scRNA-seq datasets are summarized in Supplementary Tables S18 and S19. From Supplementary Table S18, we find that for Adam, Klein, Muraro, QS_Diaphragm, QS_Heart, QS_Limb_Muscle, QS_Trachea, Qx_Bladder, Qx_Limb_Muscle and Romanov, scBGEDA performs better than other algorithms in terms of NMI, with significant differences between scBGEDA and the other different algorithms (P<0.05). For Bach and Qx_Spleen, scziDesk outperforms other algorithms with significant differences, while for Tosches_turtle, there is significant difference between DESC and the other compared algorithms. We find that scBGEDA was able to significantly improve upon other methods with a rate of 0.65; while other methods were significantly better with a frequency of 0.35 (using the parameters optimized for the 20 datasets). From Supplementary Table S19, we observe that there are significant differences for ARI values between scBGEDA and the other clustering algorithms on nine scRNA-seq datasets (P<0.05), including Adam, Muraro, QS_Diaphragm, QS_Heart, QS_Limb_Muscle, QS_Trachea, Qx_Bladder, Qx_Limb_Muscle and Romanov. It demonstrates the superiority of the proposed algorithm scBGEDA.

To compare intuitively the discrimination ability of scBGEDA to the four other deep learning-based algorithms, two-dimensional visualizations were plotted on five scRNA-seq datasets, including Adam, Klein, Muraro, QS_Trachea and Qx_Limb_Muscle. First, we obtained the feature representation of the scRNA-seq data from the latent space with 32 dimensions. Then, we applied the Uniform Manifold Approximation and Projection (UMAP) dimension reduction method to visualize the embedded data in a 2D plane using the default parameters. Figure 2D summarizes the visual results using the five scRNA-seq datasets. From the figure, we see that scBGEDA clustering results in almost no overlap between cell types, indicating that scBGEDA clearly distinguishes the cell groups in a 2D plane for both simple and complex scRNA-seq datasets. The other clustering methods, especially DCA and DESC, fail to partition cells into correct cell clusters, indicating that scBGEDA is superior to other scRNA-seq clustering methods in separating similar cells. Moreover, we also reveal that our proposed method achieves a more discriminative latent representation to separate cells in a visual perspective. In summary, from different angles, we observed that the proposed scBGEDA presents competitive clustering performance compared with other single-cell clustering algorithms on simpler and more complex scRNA-seq datasets of various cell types.

### 3.4 Effects of different numbers of highly variable genes on scBGEDA

**Fig. 3.**
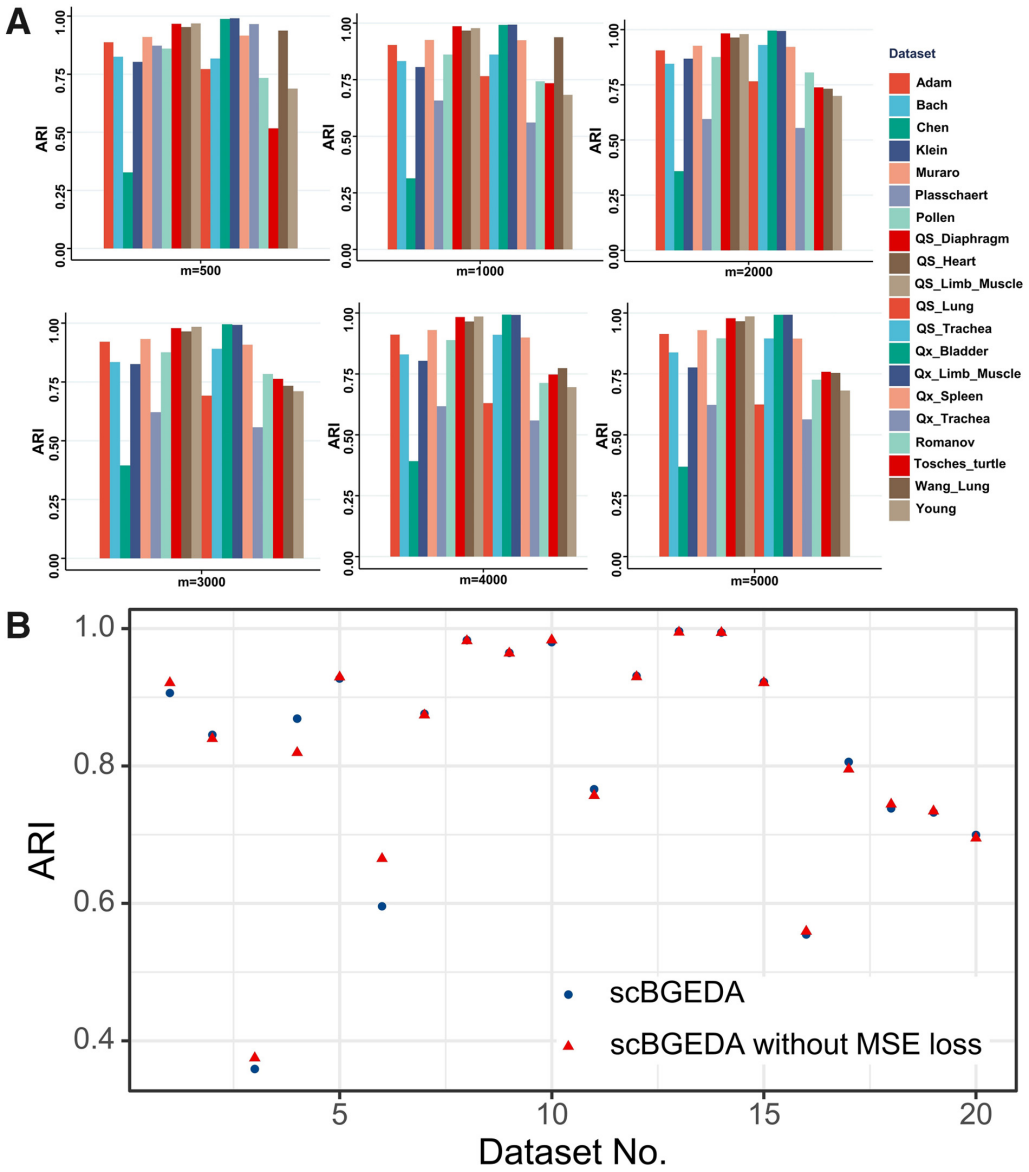
(**A**) Bar plot of ARI values measuring scBGEDA with different numbers of highly variable genes. (**B**) Clustering result comparison of scBGEDA with or without MSE loss measured by ARI

For this experiment, to test the effect of input number of highly variable genes, several highly variable genes were set as input features for the dual denoising autoencoder network in scBGEDA. Indeed, different numbers of highly variable genes could have dissimilar effects on the clustering performance of scBGEDA. Taking too many genes as input features in the model could lead to a slow running speed and a high memory requirement. Using only a few genes may lead to multiple informative genes being dropped and the remaining genes not covering all the dataset, resulting in low-quality clustering. To investigate the effect of different numbers of highly variable genes (*m*), we varied them as within {500,1000,2000,3000,4000,5000} and tested this on the 20 real scRNA-seq datasets. The experimental results expressed as ARI values are summarized in Figure 3A. We observe that the model with m=2000 is superior to all other models {500,1000,3000,4000,5000} on 16, 14, 9, 9 and 10 scRNA-seq datasets, respectively. Specifically, the model with m=2000 achieves the best clustering results on six scRNA-seq datasets, Klein, Bach, Qx_Bladder, Qx_Limb_Muscle, QS_Trachea and Romanov and while the other models provide best clustering performances on at most three scRNA-seq datasets. Therefore, m=2000 was chosen for the scBGEDA model, which is also consistent with the analysis of the number of highly variable genes in scBGEDA.

### 3.5 Effects of MSE loss

In our proposed scBGEDA, the dual denoising autoencoder network adopts a double loss function to learn the latent representation, which synergistically optimizes ZINB reconstruction loss and denoising MSE loss. To explore the importance of MSE loss on clustering performance, we compared scBGEDA with and without MSE loss on the 20 real scRNA-seq datasets by ARI metric. We summarize the clustering results in Figure 3B and Supplementary Table S20. From the figure, we see that complete scBGEDA obtains a better clustering performance than scBGEDA without MSE loss on most datasets, showing that a dual denoising autoencoder structure with dual loss function often (but not always) improves performance. In summary, we conclude that the MSE loss in the autoencoder brings a positive effect on clustering performance in scBGEDA.

### 3.6 Running time comparison of scBGEDA with other deep-learning methods

**Fig. 4.**
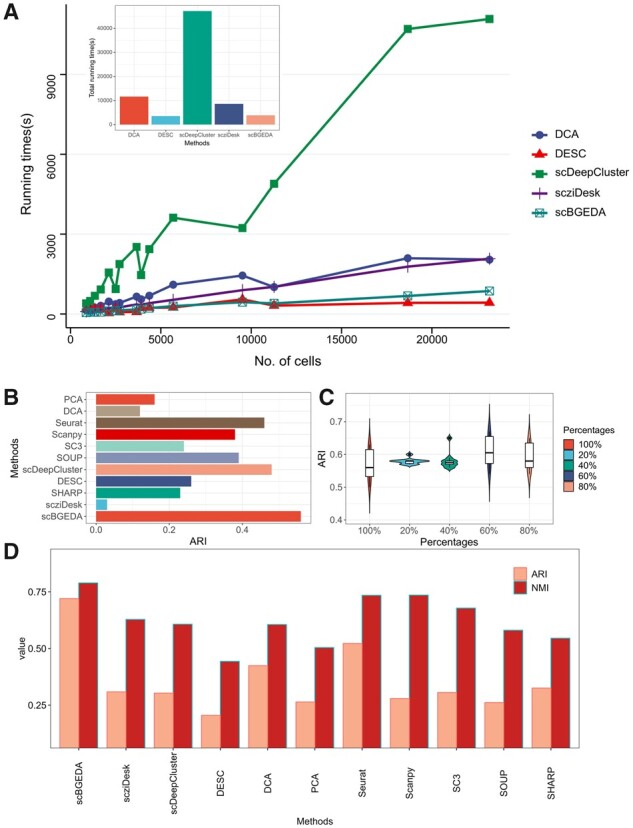
(**A**) The running time and total time cost comparisons of different deep learning-based models on specific datasets of varying cell size. (**B**) Results of comparative analysis of different clustering methods on the large-scale scRNA-seq dataset PBMC_68k measured by ARI. (**C**) The change of ARI values from whole data to 20%, 40%, 60% and 80% downsampling data in PBMC_68k dataset. (**D**) Comparison results for scBGEDA and different clustering methods on ‘Tabula Muris’ measured by NMI and ARI

The running time of the proposed scBGEDA was investigated compared with four deep learning-based algorithms, including DCA, DESC, scDeepCluster and scziDesk, on the 20 real scRNA-seq datasets. We summarize the running times of the different computational algorithms on the 20 datasets in Figure 4A. From the figure, we observe that the time complexity of our proposed algorithm, scBGEDA and the scziDesk algorithm, is nearly linear with the increasing number of cells, however the slope of the scBGEDA plot is lower, meaning that scBGEDA has higher computational power than scziDesk on very large scRNA-seq datasets. DESC, a soft clustering algorithm with useful cluster assignment probabilities, has a slightly lower time cost for larger scRNA-seq datasets containing more than 10 000 cells. DCA and scDeepCluster are more time consuming than scBGEDA on scRNA-seq datasets of different cell sizes. Due to the early stopping mechanism, the time trend curves of DCA and scDeepCluster show substantial fluctuation. Moreover, from the total times of all datasets summarized in Figure 4A, scBGEDA surpasses scziDesk and scDeepCluster and although the total time cost of DESC is slightly lower than scBGEDA, DESC does not obtain the desired clustering result. In summary, we can conclude that scBGEDA has an appropriate running time and is an efficient tool in variable size scRNA-seq data analysis.

### 3.7 Evaluations on two large-scale scRNA-seq datasets

To demonstrate the scalability of scBGEDA to large-scale scRNA-seq data, several experiments were conducted on two large-scale scRNA-seq datasets, PBMC_68k (Zheng *et al.*, 2017) and ‘Tabula Muris’ (Tabula Muris Consortium *et al.*, 2018). PBMC_68k has 68 000 peripheral blood mononuclear cells with 10 cell types including Activated CD8+; Naive CD8+; Memory and Reg T cells; Naive CD4+; NK; Naive CD8+; B; Megakaryocytes; Monocytes and dendritic cells; and B, dendritic, T cells. ‘Tabula Muris’ has nearly 100 000 cells from 20 organs and tissues and 19 179 genes with 55 cell types. We compared the proposed scBGEDA with six scRNA-seq clustering algorithms and four deep learning-based models, SHARP, SOUP, Seurat, SC3, Scanpy, PCA, DCA, scDeepCluster, scziDesk and DESC. CIDR was not chosen as it takes more than 141G memory to run on PBMC_68k and ‘Tabula Muris’. The comparison results on PBMC_68k measured by ARI, NMI and Wilcoxon test analysis are summarized in Figure 4B, Supplementary Figure S2 and Supplementary Table S21, respectively. We see that our proposed method outperforms the other clustering methods. Moreover, from Supplementary Table S9, we find significant differences between scBGEDA and the other algorithms on the large-scale scRNA-seq dataset PBMC_68k (P<0.05), further confirming that scBGEDA performs well for clustering tasks on larger scRNA-seq datasets. Moreover, Figure 4D illustrates the clustering performance of scBGEDA measured by NMI and ARI compared to the other clustering algorithms on ‘Tabula Muris’. As shown in Figure 4D, it further demonstrates that scBGEDA can provide excellent clustering performance on the large-scale scRNA-seq dataset.

To further validate the robustness of scBGEDA, we downsampled the PBMC_68k dataset to yield partial datasets containing 20%, 40%, 60% and 80% of the cells from the whole data. The same 10 random seeds were used to select the cells randomly to ensure fairness. The median ARI values out of 10 runs are summarized in Figure 4C. We observe that scBGEDA performs excellently on each dataset size. Besides, the Wilconxon test result on the ARI results is summarized in Supplementary Table S22, showing there is no significance difference between the ARI values of scBGEDA on different data sizes of PMBC_68k (P>0.05), indicating the robustness of scBGEDA.

### 3.8 Distribution analysis

To explore the suitable distribution in modeling the scRNA-seq data, we apply the NB and ZINB models in our proposed algorithm scBGEDA on those 20 scRNA-seq datasets. To conduct a fair comparison, we replaced the ZINB distribution in the scBGEDA algorithm with the NB distribution. The performance comparison is summarized in Supplementary Figure S3, measured by NMI and ARI. From Supplementary Figure S3, we can observe that scBGEDA with NB or ZINB models shows comparable clustering results, with the average NMI and ARI of scBGEDA with NB model just slightly higher than those of scBGEDA with ZINB model. In particular, for the Adam, Klein, Muraro, Plasschaert, Pollen, QS_Heart, QS_Limb_Muscle, Qx_Limb_Muscle, Tosches_turtle, Wang_Lung and Young datasets, scBGEDA with the NB model performs better than scBGEDA with ZINB.

### 3.9 Trajectory inference

**Fig. 5.**
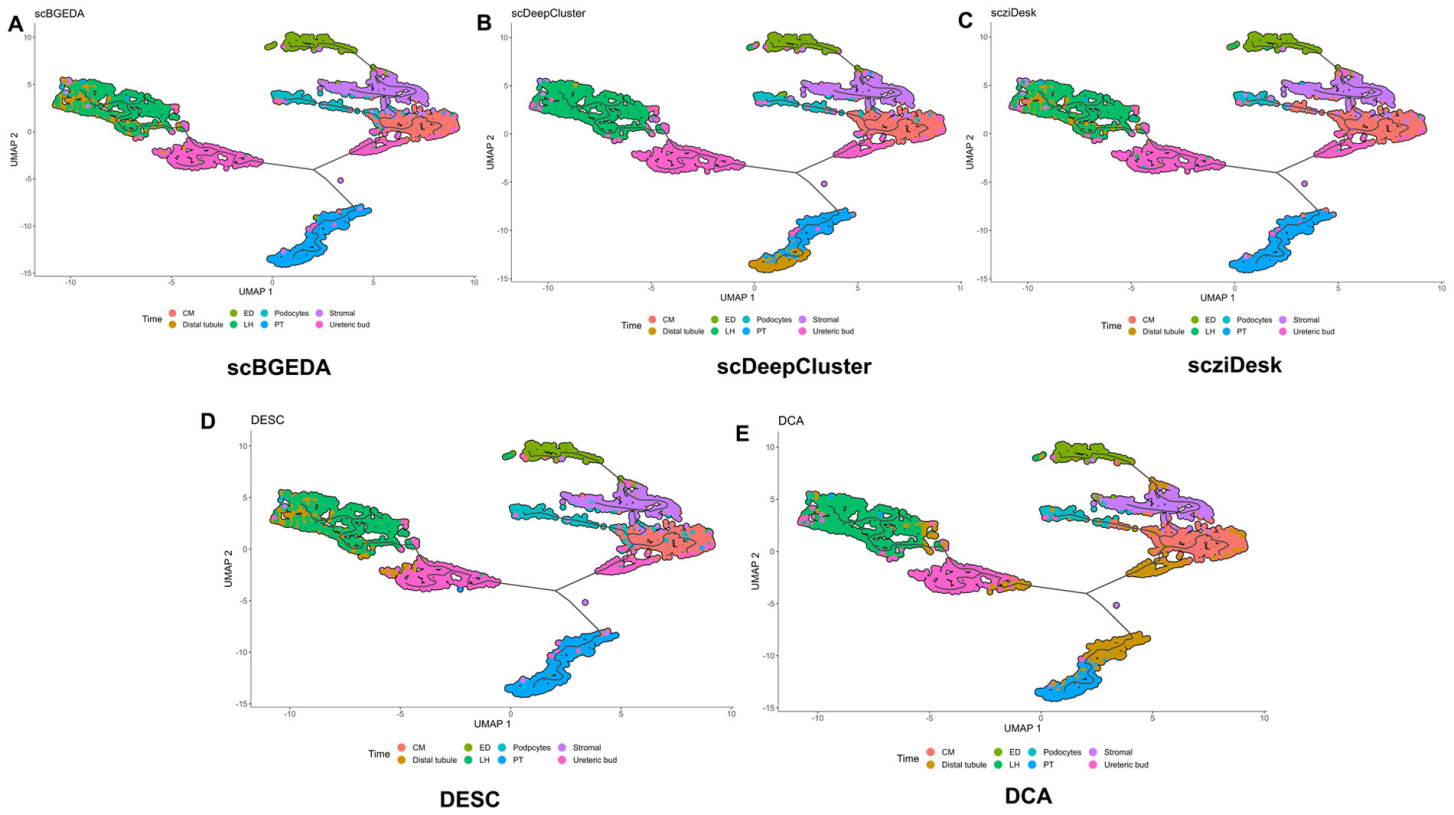
UMAP visualization of the trajectory inference on ‘Adam’ by the different computation methods. Black lines represent branched cell trajectories, and cell types are different colors

To demonstrate the performance of the different computational methods for trajectory inference, we applied Monocle3 (Cao *et al.*, 2019) for the gene expression data and prediction labels obtained by the different computational methods. The experimental results for the trajectory inference are summarized in Figure 5, where we can observe that our proposed scBGEDA and scziDesk both produce a more accurate order of pseudotime of kidney development from early proximal tubule progenitor cell to the two major subgroups. One of the subgroups is the loop of Henle (left branch), which leads from the proximal convoluted tubule to the distal convoluted tubule and represents a more mature developmental stage. The other subgroup (right branch) has endothelial cell populations, which have extensive diversity in the kidney. We notice that ureteric buds can be seen in both branches. This is consistent with the fact that ureteric bud appears during the embryological development of kidney (Adam *et al.*, 2017).

### 3.10 Ablation analysis

**Fig. 6.**
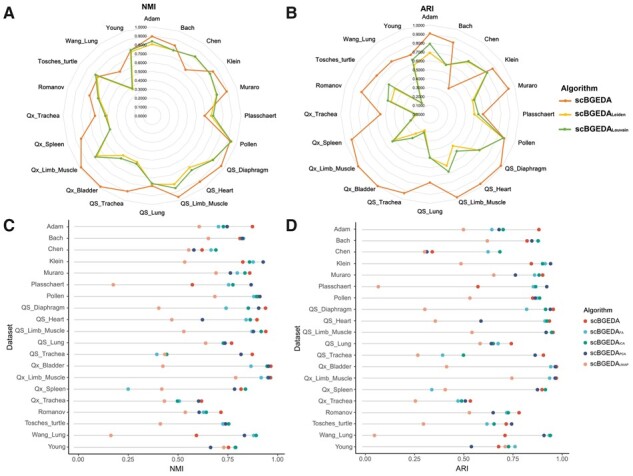
Ablation analysis results. (**A and B**) Comparative performance of scBGEDA with different clustering algorithms measured by NMI and ARI; (**C and D**) Comparative performance of scBGEDA with different dimension reduction algorithms measured by NMI and ARI

In this experiment, we analyzed the impact of each component of scBGEDA. The single-cell dual denoising autoencoder and the bipartite graph ensemble clustering were ablated and the model tested on the 20 scRNA-seq datasets. (i) We removed the bipartite graph ensemble clustering in scBGEDA, and investigated the latent feature representation provided by the single-cell dual denoising autoencoder with the regular clustering including the Leiden and Louvain clustering (Wolf *et al.*, 2018), called scBGEDA${}_{\mathrm{Leiden}}$ and scBGDDA${}_{\mathrm{Louvain}}$, respectively; (ii) we removed the single-cell dual denoising autoencoder in scBGEDA, and applied the proposed bipartite graph ensemble clustering on a reduced-dimensional space with 25 dimensions obtained by PCA (Fodor, 2002), factor analysis (FA) (Fodor, 2002) and UMAP (McInnes *et al.*, 2018), called scBGEDA${}_{\mathrm{PCA}}$, scBGEDA${}_{FA}$ and scBGEDA${}_{\mathrm{UMAP}}$, respectively. The experimental results are summarized in Figure 6 and Supplementary Tables S23 and S24 measured by NMI, ARI, ASW and cLISI. We observe that the synergistic use of a single-cell dual denoising autoencoder and the bipartite graph ensemble clustering often (but not always) enhance the clustering performance. As observed from Figure 6C and D, we find that for most scRNA-seq datasets, the single-cell dual denoising autoencoder improves performance with respect to other competitor methods (by typically a small amount). While for some scRNA-seq datasets including Chen, Klein, Plasschaert, Tosches_turtle, Wang_Lung and Young, the single-cell dual denoising autoencoder can significantly reduce performance as well. Each component of scBGEDA plays an important role in characterizing scRNA-seq data. Moreover, to assess different dimension reduction models, including the AE in our proposed scBGEDA, UMAP, PCA and FA. The nearest neighbor error (NNE) (Pouyan and Kostka, 2018) was employed to measure these dimension reduction methods. NNE is calculated using a nearest neighbor classifier based on the reduced-dimensional space to be evaluated, which can reflect the goodness of the distance measure from the latent features directly. Predictions for each cell were obtained using 10-fold cross-validation (9 for training and 1 for validation), and the proportion of misclassified cells was reported by NNE (Wang *et al.*, 2017). Accordingly, we report the average over 20 runs of the average validation error from the 10 folders as the final NNE error. The NNE obtained from the methods is summarized in Supplementary Figure S4. As can be seen from this figure, the latent potential features acquired by the proposed AE are comparable to PCA and superior to UMAP and FA in the comparison of the reduced-dimensional space of the various approaches.

### 3.11 scBGEDA assists marker gene identification

**Fig. 7.**
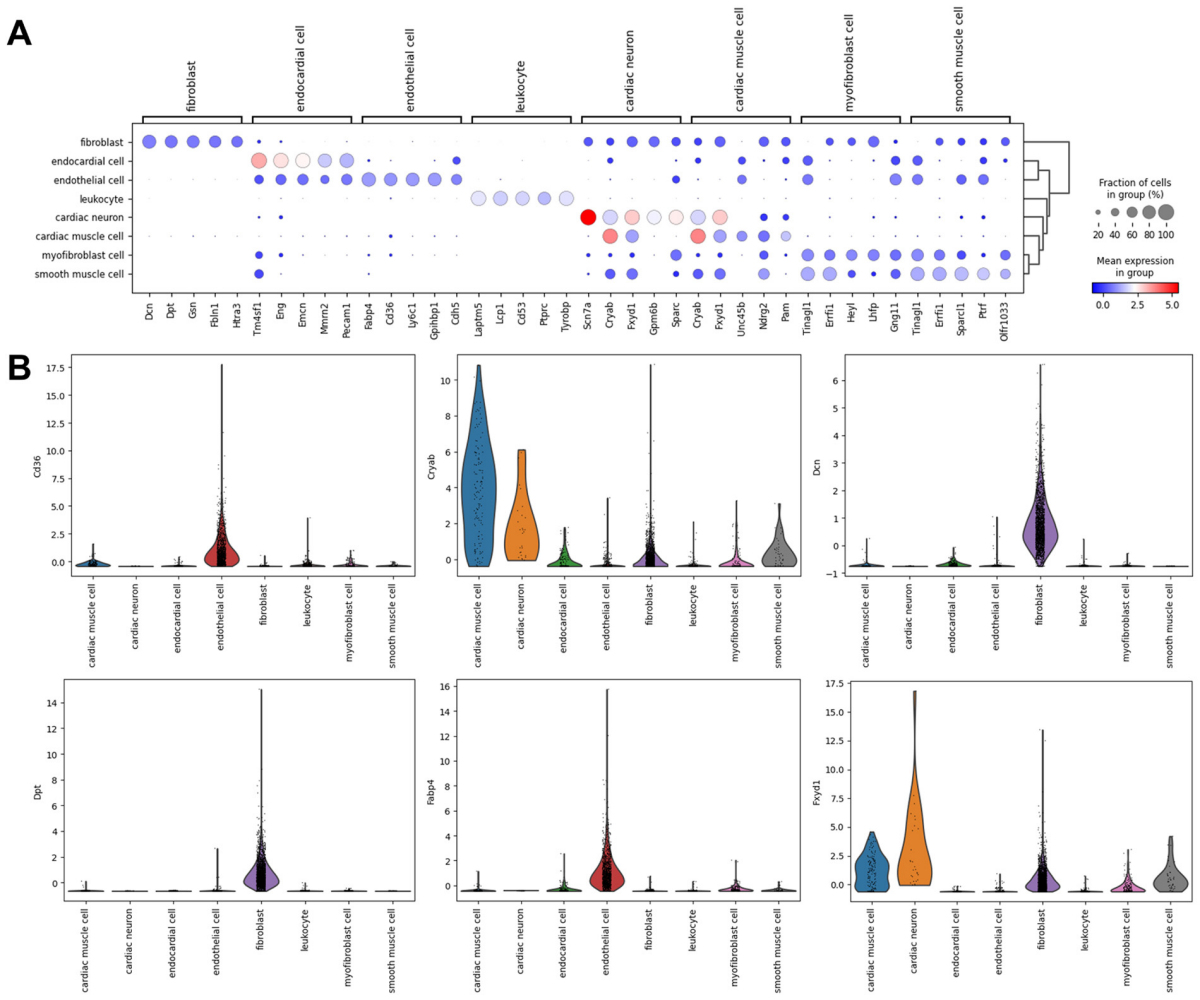
Marker gene analysis in the scRNA-seq dataset QS_Heart. (**A**) Dot plot of average expression of the top five DEGs within each cell type, implying marker genes of QS_Heart. (**B**) The violin plot of identified marker gene expression in the different cell types

We employed the gene expression matrix of the QS_Heart dataset and took the predicted clustering labels of scBGEDA to identify differentially expressed genes (DEGs) and thereby, the marker genes for each cluster. First, we conducted differential expression analysis to determine the DEGs in each cluster using the Wilcoxon Ranksum test, to ascertain whether two independent cell types are from the same distribution in a non-parametric form. To identify the predominantly expressed genes in each cluster, the top 20 DEGs with *P*-values <0.05 were reported as shown in Supplementary Figure S5 that drive the expression–distribution separation of the different cell clusters. Then, we visualized the expression levels of the top five genes in each cluster to observe the expression levels of the highly expressed genes in each cluster. Figure 7A and Supplementary Figure S6 indicate the average expression of each of the top five DEGs of each cluster. To verify the obtained marker genes, we matched them manually to the published marker genes in the cell marker database CellMark (Zhang *et al.*, 2019). It can be seen that most of the DEGs identified by scBGEDA can be matched to published marker genes within the clusters; for instance, Gsn, Col3a1, Col1a2 and Mmp2, are marker genes for fibroblast; and Fabp4, Egfl7, Flt1 and Pecam1 marker genes for endothelial cells.

It is noteworthy that although some DEGs cannot be matched to any of those in the cell marker database, they are clearly more highly expressed in some specific cell groups than in others and may perhaps indicate novel markers. For instance, in Figure 7B, the expression levels of cd36 and Fabp4 were higher in endothelial cells than in other cell types, and they are therefore potential marker genes for endothelial cells. Cryab and Fxyd1 are highly expressed in cardiac muscle and cardiac neuron cell types, respectively, and could be candidate markers for myocardium and cardiac neurons. Dcn and Dpt are highly expressed in fibroblasts, which indicates possible new markers for fibroblasts. Moreover, extended analysis including functional genomic analysis, batch effect analysis and extend experiments are summarized in Supplementary Sections S7–S9.

## 4 Conclusion

In this study, a deep single-cell clustering model via a dual denoising autoencoder with bipartite graph ensemble clustering, scBGEDA, was developed to identify cell populations in scRNA-seq datasets. Stepwise, the high-dimensional scRNA-seq data are first preprocessed and the top highly variable genes are selected to eliminate redundant genes with low expression that may disturb the clustering result. This leads to a significant improvement in clustering performance. Next, we designed a dual denoising autoencoder by optimizing the dual reconstruction loss to learn the discriminative feature representation of the scRNA-seq data. Then, we developed a bipartite graph ensemble clustering with a graph-based consensus function to identify the cell types from the learned latent representation. To validate our model, we carried out a comprehensive study comparing it to other benchmark methods in terms of cell-type identification and characterization mechanisms from different perspectives and demonstrated the superiority of scBGEDA over current methods. As the development of the advanced high-throughput technologies for scRNA-seq and the emerging cell atlas (Han *et al.*, 2018; Rozenblatt-Rosen *et al.*, 2017), we will explore the performance of the proposed scBGEDA on larger scale scRNA-seq datasets in the future.

## Funding

The work described in this article was substantially supported by the National Natural Science Foundation of China. [62206086, 62076109 and 61972174] and also funded by ‘the Fundamental Research Funds for the Central Universities’.

*Conflict of Interest*: none declared.

## Supplementary Material

btad075_Supplementary_Data

## Data Availability

The data underlying this article are available in the article and in its online supplementary material.
