# Supplementary material for: scBGEDA: deep single-cell clustering analysis via a dual denoising autoencoder with bipartite graph ensemble clustering
Source: Bioinformatics. 2023 Feb 3;39(2):btad075. doi: 10.1093/bioinformatics/btad075 (PMC9925104; doi:10.1093/bioinformatics/btad075)
Supplement: btad075_Supplementary_Data [file btad075_supplementary_data.pdf]

Supplementary Data: scBGEDA: Deep Single-cell Clustering Analysis via a Dual Denoising Autoencoder with Bipartite Graph Ensemble Clustering

Yunhe Wang, Zhuohan Yu, Shaochuan Li, Chuang Bian, Yanchun Liang, Ka-Chun Wong, Xiangtao Li*

1. Time Complexity Analysis

Generally, the time complexity of scBGEDA comprises of two components: the single-cell dual denoising autoencoder and the ensemble clustering framework. The time complexity of the autoencoder is $O(nI^2)$ [1], where n is the number of samples in the scRNA-seq dataset, and I is the maximum number of neurons in hidden layers. Considering the ensemble clustering framework, the time cost mainly lies in the generation of basic clusterings and the consensus function. The first phase costs $O(ndk_ct)$ [2], where d is the dimension, k_c is the total number of clusters in those basic clusterings, and t is the number of iterations in the *K-means* clustering method; the second phase takes $O(n(k_c + K + k^2t) + k_c^3)$, where K is the number of basic clusterings, k is the number of clusters in the clustering result. Therefore, the total time complexity of the ensemble clustering is $O(nk_c(dt + 1) + n(K + k^2t) + k_c^3)$. In conclusion, the overall time complexity of scBGEDA is $O(n(I^2 + k_cdt + k_c) + n(K + k^2t) + k_c^3$.

2. Compare using different numbers of cluster in scBGEDA

To investigate the effect of the true cluster number k on the performance of the proposed model scBGEDA, the manual cluster number perturbations are added to five scRNA-seq datasets from the Smart-seq2 platform, including QS_Diaphragm, QS_Heart, QS_Limb_Muscle, QS_Lung, and QS_Trachea. Assuming the true number of clusters is k, the experimental number of clusters is set to $\{k-2, k-1, k, k+1, k+2\}$. The experimental results measured by NMI and ARI are summarized in Supplementary Fig. S7 (A) and (B). As shown in those figures, the clustering performance of scBGEDA is necessarily based on the appropriate cluster number. In particular, for the scRNA-seq dataset with the small number of cluster, such as ”QS_Trachea”, the cluster number shows a greater impact on the performance of scBGEDA than those with the large cluster number. This is reasonable since reducing or increasing the cluster number would disrupt the structure of the scRNA-seq data that contains only two or three cell types. In summary, the cluster number plays an important role in the performance of scBGEDA.

3. Robustness to the number of hidden layers in scBGEDA

The number of hidden layers is an indispensable parameter in the autoencoder network, which can affect the learning performance of feature representations in scBGEDA. To discuss the robustness of our scBGEDA model to the number of hidden layers, three autoencoder architectures including scBGEDA([Input_layer]-256-32-256-[Output_layer]), scBGEDA1 ([Input_layer]-256-128-32-128-256-[Output_layer]), and scBGEDA2 ([Input_layer]-256-128-64-32-64-128-256-[Output_layer]) are tested on those five scRNA-seq datasets from the Smart-seq2 platform. Since the decoder has hidden layers as opposed to the encoder, we focus on the number of hidden layers in the autoencoder architecture. The comparison performances of different architectures on those scRNA-seq datasets are reported in Supplementary Fig. S7 (C) and (D) using NMI and ARI values. From Supplementary Fig. S7 (C) and (D), we can observe that scBGEDA provides consistent performance with different autoencoder architectures and scBGEDA with two hidden layers obtains a slightly better overall performance than the other architectures, demonstrating our two hidden layers in scBGEDA can optimize different deep neural network architectures effectively. Therefore, considering the relative higher clustering results, the architecture with two hidden layers in the encoder are employed in scBGEDA.

4. The impact of the dimensionality of latent feature in scBGEDA

This section discusses the impact of our scBGEDA model on different dimensions of the latent space. We use dimensions from $[16, 32, 48, 64]$. scBGEDA was performed on these 20 scRNA-seq datasets in those different dimensions. We summarize the performances in Supplementary Table S2 measured by different metrics including NMI, ARI, ASW, and cLISI. From Supplementary Table S2, we observe that scBGEDA provides comparable clustering results with different dimensions and the average metric values of a latent feature space of 32 dimensions are the highest. Therefore, in our study, we adopt 32 as the dimensionality of the latent feature space in scBGEDA.

5. Clustering Evaluation metrics

Four evaluation criteria are employed to measure the performance of each method, including the Normalized Mutual Information (NMI) [15], the Adjusted Rand Index (ARI) [16], and two biological metrics (the cell-type Average Silhouette Width (ASW) and the Cell-type Local Inverse Simpson's Index metrics (cLISI) score) [17].

In terms of NMI and ARI, NMI is widely used for measuring the consistency between the obtained clustering label and the true label, while ARI is commonly employed in evaluating the concordance between two clustering labels. Given the predicted clustering label L_e and the true clustering label L_t, NMI and ARI can be defined as follows:

$$NMI(L_e, L_t) = \frac{\sum_{e,t} n_{e,t} log \frac{n \cdot n_{e,t}}{n_e \cdot n_t}}{\sqrt{(\sum_e n_e log \frac{n_e}{n})(\sum_t n_t log \frac{n_t}{n})}} \tag{1}$$

$$ARI(L_e, L_t) = \frac{\sum_{e,t} \binom{n_{e,t}}{2} - \sum_e \binom{n_e}{2} \cdot \sum_t \binom{n_t}{2} / \binom{n}{2}}{\sum_e \binom{n_e}{2} / 2 + \sum_t \binom{n_t}{2} / 2 - \sum_e \binom{n_e}{2} \cdot \sum_t \binom{n_t}{2} / \binom{n}{2}} \tag{2}$$

where n denotes the single cell number in the dataset; n_e is the number of single cells in the cluster e of L_e, n_t is the number of single cells in the cluster t of L_t, and $n_{e,t}$ represents the number of shared single cells between the cluster e and the cluster t.

In temrs of ASW and cLISI, ASW is widely used to evaluate the data integration output, which is calculated on the embeddings obtained by integration approaches or the PCA of expression matrices for the feature output. It determines the separation of clusters, ranging from -1 to 1, where -1 or 0 denotes overlapping clusters; 1 denotes well-separated and dense clusters. cLISI is based on a diversity score (Local Inverse Simpson's Index metrics, LISI), which is computed from the neighborhood lists per node provided from the integrated single-cell kNN graphs. It ranges from 0 to 1, where 0 represents a low cell-type separation and 1 represents a perfect separation. The details of these two metrics can be found at https://github.com/theislab/scib.

Each clustering evaluation metric has its advantages and disadvantages. For NMI and ARI, one advantage of NMI is that we can use it to compare different clustering models that have different numbers of clusters because NMI is normalized. However, NMI still prefers to have a clustering result for each class of the target. One advantage of ARI is that it corrects the lack of a constant value in the Rand index when the clustering labels are chosen randomly. However, ARI is preferred when there are large equal-sized clusters [18], [19], [20]. In addition, both NMI and ARI require ground truth labels as prior knowledge. For ASW and cLISI, ASW is commonly used to determine cluster separation, and cLISI is a diversity score to evaluate cell-type separation. However, both ASW and cLISI are employed as label preservation metrics and cLISI is not applicable to graph-based outputs [17].

6. Hyperparameter selection discussion for deep learning-based competitors

For scziDesk, the hyperparameters have been optimized in [21] on the same twenty scRNA-seq datasets. For DCA, scDeepCluster, and DESC, the hyperparameters to be optimized including the numbers of highly variable genes ranging from $[500, 1000, 2000, 3000, 4000, 5000]$ and the number of hidden layers chosen from ([Input layer]-256-32-256-[Output layer]), ([Input layer]-256-128-32-128-256-[Output layer]), and ([Input layer]-256-128-64-32-64-128-256-[Output layer]) on those twenty scRNA-seq datasets. The experimental results measured by NMI, ARI, ASW, and cLISI are summarized in Supplementary Tables 3-11.

7. Functional genomic analysis

We conducted an enrichment analysis of the QS_Heart dataset to examine the effectiveness of scBGEDA in functional aspects. First, the mutual information-based feature selection of [22] was employed to choose the top 200 genes in the gene expression matrix of QS_Heart and the corresponding predicted labels provided by scBGEDA. This yielded 5202 enriched gene ontologies (GOs), including 4256 enriched GO biological processes, 385 cellular components, and 561 molecular functions.

Supplementary Fig. S8 illustrates the top 20 categories of gene ontology (GO) enrichment sorted by p-value. Especially, the top three enriched GO biological processes are extracellular structure organization (GO:0043062), extracellular matrix organization (GO:0030198), and tube development (GO: 0035295). We find that most of the first five enriched biological processes are related to the vasculature, with central relation to the heart. Further, the top three enriched GO cellular components are extracellular matrix (GO:0031012), collagen-containing extracellular matrix (GO:0062023), and extracellular region part (GO:0044421). The top three enriched GO molecular functions are extracellular matrix structural constituent (GO:0005201), glycosaminoglycan binding (GO:0005539), and collagen binding (GO:0005518). Supplementary Fig. S9 depicts the relevant gene ontology distribution. In the majority of cases, the same genes appear in more than one term; such as, of biological processes, are also expressed in "cellular process" (16906, 80.87%) and "biological regulation" (13255, 63.4%); for the cellular component category, most genes are assigned to "cell" (17555, 83.84%) and "cell part" (17555, 83.84%). For the molecular functions, the majority of genes are matched to "binding" (14872, 71.92%) and "catalytic activity" (5841, 28.25%).

Supplementary Fig. S10 summarizes a circular visualization of the gene annotation enrichment analysis for biological processes, cellular components, and molecular functions.

Moreover, to capture the relationships between the enriched terms, we entered the 200 most highly expressed genes in Metascape [23] to produce a similarity network of the terms. The network graph shown in Supplementary Fig. S11 (A) consists of a subset of enriched terms presented visually in Cytoscape [24], where terms with a similarity greater than 0.3 are connected by edges and each node represents an enriched term. Moreover, to predict and classify the possible functions of the underlying genes, we searched for the genes in the KOG database. Based on this, 153 pathways could be matched from those 9221 unigenes. Most of those genes are in ”Metabolic pathways” (1606, 17.42%) and ”Olfactory transduction” (1199, 13%), followed by ”Pathways in cancer” (551, 5.98%) and ”PI3K-Akt signaling pathway” (499, 5.41%). Supplementary Fig. 5 (B) summarizes the top 20 KEGG enrichments from p-value. The pathways were successfully annotated and divided into six groups including Metabolism, Genetic Information Processing, Environmental Information Processing, Cellular Processes, Organismal Systems, and Human Diseases as depicted in Supplementary Fig. S11 (C). We also show in Supplementary Fig. S12 the three levels of KEGG functional categories and the number of enriched genes for the top 50 KEGG pathways sorted by p-value.

In addition, we identified Fluid shear stress and atherosclerosis pathway (ko05418) from the KEGG enrichment analysis and the 11 related genes, as shown in Supplementary Fig. S13. This Fluid shear stress and atherosclerosis pathway belongs to the Cardiovascular disease. We find that Shear stress and atherosclerosis pathway are closely associated with the heart. In particular, Shear stress denotes the frictional force exerted by blood flow on the endothelial surface of the vessel wall, which is responsible for the development of atherosclerosis, playing a key role in vascular pathobiology [25], [26].

8. Batch effect analysis

To test whether our proposed algorithm scBGEDA can remove batch effect, we employ four publicly available human pancreas datasets using Fluidigm C1, SMART-seq2, CEL-seq, and CEL-seq2 [27]. To show the effectiveness of our model, we compared scBGEDA with three standard batch effect correction methods, including Combat [28], Harmony [29], and BBKNN [30] on that dataset. The comparison results are summarized in Supplementary Fig. S14 in the supplementary material. As depicted in this figure, we observe that our proposed model can successfully remove all batches effect, which is similar to the results of Combat and Harmony, yet better than BBKNN. In general, our proposed algorithm has the potential to eliminate batch effect.

9. Additional analysis results

On the one hand, we have followed your comments to conduct scBGEDA with the default experiment settings of other deep learning-based models including scziDesk, scDeepCluster, DCA, and DESC (the data preprocessing step and the dimensionality of latent representation), called scBGEDA$_{sczi}$, scBGEDA$_{scDeep}$, scBGEDA$_{DCA}$, and scBGEDA$_{DESC}$ respectively. The experimental results measured by NMI, ARI, ASW, and cLISI, are summarized in Supplementary Fig. S15. As can be seen from this figure, scBGEDA performs better than those containing other experimental settings of particular deep learning-based algorithms.

On the other hand, to demonstrate the performance of our clustering step compared to baseline methods, we have integrated the clustering step of scBGEDA (Bipartite Graph Ensemble Clustering, BGEC) into other deep learning-based models to make a fair comparison. The experimental results measured by different metrics including NMI, ARI, ASW, and cLISI are summarized in Supplementary Tables S12-S13. From Supplementary Tables S12-S13, we observe that scBGEDA is better than other deep learning-based algorithms containing BGEC clustering, further indicating the effectiveness of the proposed scBGEDA with a fair approach.

References

[1] X. Guo, L. Gao, X. Liu, and J. Yin, "Improved deep embedded clustering with local structure preservation." in *Ijcai*, 2017, pp. 1753–1759.

[2] D. Huang, C.-D. Wang, J.-S. Wu, J.-H. Lai, and C.-K. Kwoh, "Ultra-scalable spectral clustering and ensemble clustering," *IEEE Transactions on Knowledge and Data Engineering*, vol. 32, no. 6, pp. 1212–1226, 2019.

[3] M. Adam, A. S. Potter, and S. S. Potter, "Psychrophilic proteases dramatically reduce single-cell rna-seq artifacts: a molecular atlas of kidney development," *Development*, vol. 144, no. 19, pp. 3625–3632, 2017.

[4] K. Bach, S. Pensa, M. Grzelak, J. Hadfield, D. J. Adams, J. C. Marioni, and W. T. Khaled, "Differentiation dynamics of mammary epithelial cells revealed by single-cell rna sequencing," *Nature communications*, vol. 8, no. 1, pp. 1–11, 2017.

[5] R. Chen, X. Wu, L. Jiang, and Y. Zhang, "Single-cell rna-seq reveals hypothalamic cell diversity," *Cell reports*, vol. 18, no. 13, pp. 3227–3241, 2017.

[6] A. M. Klein, L. Mazutis, I. Akartuna, N. Tallapragada, A. Veres, V. Li, L. Peshkin, D. A. Weitz, and M. W. Kirschner, "Droplet barcoding for single-cell transcriptomics applied to embryonic stem cells," *Cell*, vol. 161, no. 5, pp. 1187–1201, 2015.

[7] M. J. Muraro, G. Dharmadhikari, D. Grün, N. Groen, T. Dielen, E. Jansen, L. Van Gurp, M. A. Engelse, F. Carlotti, E. J. De Koning *et al.*, "A single-cell transcriptome atlas of the human pancreas," *Cell systems*, vol. 3, no. 4, pp. 385–394, 2016.

[8] L. W. Plasschaert, R. Žilionis, R. Choo-Wing, V. Savova, J. Knehr, G. Roma, A. M. Klein, and A. B. Jaffe, "A single-cell atlas of the airway epithelium reveals the cftr-rich pulmonary ionocyte," *Nature*, vol. 560, no. 7718, pp. 377–381, 2018.

[9] A. A. Pollen, T. J. Nowakowski, J. Shuga, X. Wang, A. A. Leyrat, J. H. Lui, N. Li, L. Szpankowski, B. Fowler, P. Chen *et al.*, "Low-coverage single-cell mrna sequencing reveals cellular heterogeneity and activated signaling pathways in developing cerebral cortex," *Nature biotechnology*, vol. 32, no. 10, pp. 1053–1058, 2014.

[10] N. Schaum, J. Karkanias, N. F. Neff, A. P. May, S. R. Quake, T. Wyss-Coray, S. Darmanis, J. Batson, O. Botvinnik, M. B. Chen *et al.*, "Single-cell transcriptomics of 20 mouse organs creates a tabula muris: The tabula muris consortium," *Nature*, vol. 562, no. 7727, p. 367, 2018.

[11] R. A. Romanov, A. Zeisel, J. Bakker, F. Girach, A. Hellysaz, R. Tomer, A. Alpar, J. Mulder, F. Clotman, E. Keimpema *et al.*, "Molecular interrogation of hypothalamic organization reveals distinct dopamine neuronal subtypes," *Nature neuroscience*, vol. 20, no. 2, pp. 176–188, 2017.

[12] M. A. Tosches, T. M. Yamawaki, R. K. Naumann, A. A. Jacobi, G. Tushev, and G. Laurent, "Evolution of pallium, hippocampus, and cortical cell types revealed by single-cell transcriptomics in reptiles," *Science*, vol. 360, no. 6391, pp. 881–888, 2018.

[13] Y. Wang, Z. Tang, H. Huang, J. Li, Z. Wang, Y. Yu, C. Zhang, J. Li, H. Dai, F. Wang *et al.*, "Pulmonary alveolar type i cell population consists of two distinct subtypes that differ in cell fate," *Proceedings of the National Academy of Sciences*, vol. 115, no. 10, pp. 2407–2412, 2018.

[14] M. D. Young, T. J. Mitchell, F. A. V. Braga, M. G. Tran, B. J. Stewart, J. R. Ferdinand, G. Collord, R. A. Botting, D.-M. Popescu, K. W. Loudon *et al.*, "Single-cell transcriptomes from human kidneys reveal the cellular identity of renal tumors," *Science*, vol. 361, no. 6402, pp. 594–599, 2018.

[15] N. X. Vinh, J. Epps, and J. Bailey, "Information theoretic measures for clusterings comparison: Variants, properties, normalization and correction for chance," *The Journal of Machine Learning Research*, vol. 11, pp. 2837–2854, 2010.

[16] J. M. Santos and M. Embrechts, "On the use of the adjusted rand index as a metric for evaluating supervised classification," in *International conference on artificial neural networks*. Springer, 2009, pp. 175–184.

[17] M. D. Luecken, M. Büttner, K. Chaichoompu, A. Danese, M. Interlandi, M. F. Müller, D. C. Strobl, L. Zappia, M. Dugas, M. Colomé-Tatché *et al.*, "Benchmarking atlas-level data integration in single-cell genomics," *Nature methods*, vol. 19, no. 1, pp. 41–50, 2022.

[18] S. Romano, N. X. Vinh, J. Bailey, and K. Verspoor, "Adjusting for chance clustering comparison measures," *The Journal of Machine Learning Research*, vol. 17, no. 1, pp. 4635–4666, 2016.

[19] S. Chen, G. Yan, W. Zhang, J. Li, R. Jiang, and Z. Lin, "Ra3 is a reference-guided approach for epigenetic characterization of single cells," *Nature communications*, vol. 12, no. 1, pp. 1–13, 2021.

[20] C. Shengquan, Z. Boheng, C. Xiaoyang, Z. Xuegong, and J. Rui, "stplus: a reference-based method for the accurate enhancement of spatial transcriptomics," *Bioinformatics*, vol. 37, no. Supplement_1, pp. i299–i307, 2021.

[21] L. Chen, W. Wang, Y. Zhai, and M. Deng, "Deep soft k-means clustering with self-training for single-cell rna sequence data," *NAR Genomics and Bioinformatics*, vol. 2, no. 2, p. lqaa039, 2020.

[22] G. Brown, A. Pocock, M.-J. Zhao, and M. Luján, "Conditional likelihood maximisation: a unifying framework for information theoretic feature selection," *The journal of machine learning research*, vol. 13, pp. 27–66, 2012.

[23] Y. Zhou, B. Zhou, L. Pache, M. Chang, A. H. Khodabakhshi, O. Tanaseichuk, C. Benner, and S. K. Chanda, "Metascape provides a biologist-oriented resource for the analysis of systems-level datasets," *Nature communications*, vol. 10, no. 1, pp. 1–10, 2019.

[24] P. Shannon, A. Markiel, O. Ozier, N. S. Baliga, J. T. Wang, D. Ramage, N. Amin, B. Schwikowski, and T. Ideker, "Cytoscape: a software environment for integrated models of biomolecular interaction networks," *Genome research*, vol. 13, no. 11, pp. 2498–2504, 2003.

[25] D. Lu and G. S. Kassab, "Role of shear stress and stretch in vascular mechanobiology," *Journal of the royal society interface*, vol. 8, no. 63, pp. 1379–1385, 2011.

[26] P. N. Hopkins, "Molecular biology of atherosclerosis," *Physiological reviews*, vol. 93, no. 3, pp. 1317–1542, 2013.

[27] X. Li, K. Wang, Y. Lyu, H. Pan, J. Zhang, D. Stambolian, K. Susztak, M. P. Reilly, G. Hu, and M. Li, "Deep learning enables accurate clustering with batch effect removal in single-cell rna-seq analysis," *Nature communications*, vol. 11, no. 1, pp. 1–14, 2020.

[28] W. E. Johnson, C. Li, and A. Rabinovic, "Adjusting batch effects in microarray expression data using empirical bayes methods," *Biostatistics*, vol. 8, no. 1, pp. 118–127, 2007.

[29] I. Korsunsky, N. Millard, J. Fan, K. Slowikowski, F. Zhang, K. Wei, Y. Baglaenko, M. Brenner, P.-r. Loh, and S. Raychaudhuri, "Fast, sensitive and accurate integration of single-cell data with harmony," *Nature methods*, vol. 16, no. 12, pp. 1289–1296, 2019.

[30] K. Polański, M. D. Young, Z. Miao, K. B. Meyer, S. A. Teichmann, and J.-E. Park, "Bbknn: fast batch alignment of single cell transcriptomes," *Bioinformatics*, vol. 36, no. 3, pp. 964–965, 2020.

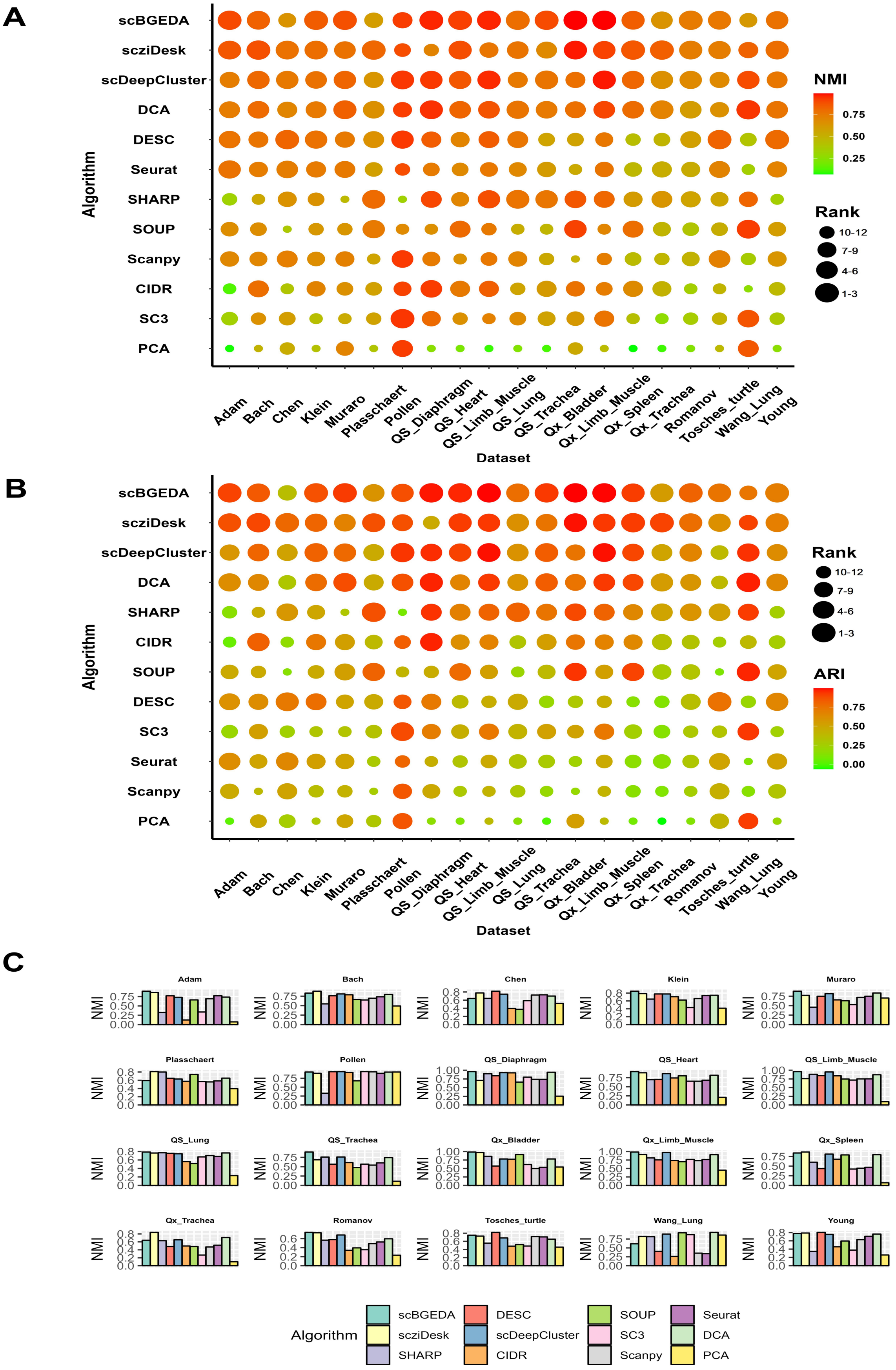

Supplementary Fig. S1: (A, B) The dot plots of NMI and ARI values of those twenty real datasets. The X-axis lists each scRNA-seq dataset and the Y-axis lists each clustering method. (C) The clustering performance comparison of different clustering algorithms on twenty real scRNA-seq datasets measured by NMI. The X-axis denotes each method and the Y-axis denotes the NMI value.

Supplementary Table S1: The characteristics of the real scRNA-seq datasets. (QS and Qx represent Quake Smart-seq2 and Quake 10x, respectively)

No.	dataset	organ	platform	cell types	cells	zero percentage	reference
1	Adam	Kidney	Drop-seq	8	3660	92.33%	[3]
2	Bach	Gammary Gland	10x	8	23184	88.04%	[4]
3	Chen	Brain	Drop-seq	46	12089	93.74%	[5]
4	Klein	Embryonic_Stem_Cell	inDrop	4	2717	65.58%	[6]
5	Muraro	Pancreas	CEL-seq2	9	2122	73.02%	[7]
6	Plasschaert	Trachea	inDrop	8	6977	92.57%	[8]
7	Pollen	Others	Unknown	11	301	64.11%	[9]
8	QS_Diaphragm	Diaphragm	Smart-seq2	5	870	91.35%	[10]
9	QS_Heart	Heart	Smart-seq2	8	4365	88.52%	[10]
10	QS_Limb_Muscle	Limb Muscle	Smart-seq2	6	1090	89.47%	[10]
11	QS_Lung	Lung	Smart-seq2	11	1676	89.08%	[10]
12	QS_Trachea	Trachea	Smart-seq2	4	1350	85.48%	[10]
13	Qx_Bladder	Bladder	10x	4	2500	86.94%	[10]
14	Qx_Limb_Muscle	Limb Muscle	10x	6	3909	93.57%	[10]
15	Qx_Spleen	Spleen	10x	5	9552	94.34%	[10]
16	Qx_Trachea	Trachea	10x	5	11269	93.66%	[10]
17	Romanov	Hypothalamus	Unknown	7	2881	85.92%	[11]
18	Tosches_turtle	turtle Brain	Drop-seq	15	18664	90.83%	[12]
19	Wang_Lung	Lung	10x	2	9519	85.31%	[13]
20	Young	Kidney	10x	11	5685	94.70%	[14]

Supplementary Table 2 Comparison performance of scBGEDA with different numbers of dimensionality of the latent feature space measured by NMI, ARI, ASW, and cLISI.

	NMI				ARI				ASW				cLISI			
Dataset	16	32	48	64	16	32	48	64	16	32	48	64	16	32	48	64
Adam	0.8930	0.8938	0.8965	0.8968	0.9080	0.9061	0.9108	0.9167	0.6287	0.6298	0.6391	0.6249	0.8951	1.0000	0.9033	0.8976
Bach	0.8149	0.8307	0.8290	0.8250	0.8329	0.8453	0.8448	0.8439	0.5367	0.5661	0.5789	0.5881	1.0000	1.0000	1.0000	0.9999
Chen	0.6491	0.6410	0.6305	0.6265	0.3818	0.3590	0.3293	0.3175	0.5646	0.5897	0.5996	0.6099	0.9941	1.0000	0.9962	0.9945
Klein	0.8228	0.8471	0.8170	0.8172	0.8270	0.8688	0.8208	0.8227	0.7323	0.7265	0.7095	0.6678	0.9994	1.0000	0.9993	0.9992
Muraro	0.8818	0.8817	0.8817	0.8817	0.9266	0.9271	0.9265	0.9277	0.5696	0.5933	0.6232	0.6256	0.9931	1.0000	0.9952	0.9955
Plasschaert	0.6013	0.5922	0.7033	0.6896	0.6054	0.5957	0.7608	0.7624	0.4841	0.4536	0.4255	0.4249	1.0000	1.0000	1.0000	1.0000
Pollen	0.9262	0.9231	0.9207	0.9222	0.8768	0.8759	0.8776	0.8716	0.7794	0.7783	0.7887	0.7851	0.9776	1.0000	0.9280	0.9335
QS_Diaphragm	0.9563	0.9611	0.9583	0.9631	0.9806	0.9830	0.9813	0.9835	0.8612	0.8772	0.8855	0.8805	0.9980	1.0000	0.9993	0.9994
QS_Heart	0.9071	0.9166	0.9154	0.9213	0.9631	0.9647	0.9646	0.9678	0.7194	0.7176	0.7333	0.7541	1.0000	1.0000	1.0000	1.0000
QS_Limb_Muscle	0.9622	0.9609	0.9609	0.9676	0.9803	0.9801	0.9811	0.9857	0.8446	0.8524	0.8573	0.8585	1.0000	1.0000	0.9999	0.9997
QS_Lung	0.7868	0.7884	0.7964	0.7988	0.7662	0.7660	0.7668	0.7635	0.5858	0.6289	0.6332	0.6269	0.9991	1.0000	0.9988	0.9981
QS_Trachea	0.8676	0.8929	0.9018	0.8592	0.9068	0.9309	0.9432	0.8994	0.6317	0.6655	0.6599	0.6447	0.9998	1.0000	1.0000	0.9999
Qx_Bladder	0.9903	0.9863	0.9880	0.9928	0.9975	0.9960	0.9964	0.9976	0.7343	0.7609	0.7790	0.7810	1.0000	1.0000	1.0000	1.0000
Qx_Limb_Muscle	0.9836	0.9864	0.9857	0.9873	0.9930	0.9941	0.9939	0.9947	0.8200	0.8262	0.8335	0.8366	1.0000	1.0000	1.0000	1.0000
Qx_Spleen	0.8074	0.8389	0.8207	0.8257	0.9201	0.9221	0.9180	0.9202	0.5958	0.6772	0.6960	0.7138	1.0000	1.0000	1.0000	1.0000
Qx_Trachea	0.6437	0.6414	0.6408	0.6420	0.5638	0.5546	0.5539	0.5571	0.4655	0.5018	0.5150	0.5182	1.0000	1.0000	1.0000	1.0000
Romanov	0.7263	0.7388	0.7300	0.7329	0.7894	0.8059	0.7978	0.8009	0.4417	0.4517	0.4583	0.4482	0.9502	1.0000	0.9330	0.9259
Tosches_turtle	0.7694	0.7510	0.7635	0.7661	0.7469	0.7383	0.7572	0.7493	0.5767	0.5998	0.6115	0.6217	0.9567	1.0000	0.9518	0.9548
Wang_Lung	0.6873	0.6151	0.6132	0.6143	0.8044	0.7323	0.7303	0.7314	0.4102	0.4380	0.4774	0.5103	1.0000	1.0000	1.0000	1.0000
Young	0.7880	0.7759	0.7693	0.7644	0.7023	0.6996	0.6928	0.6747	0.3919	0.4526	0.4479	0.4429	0.9498	1.0000	0.9400	0.9264
Average	0.8232	0.8232	0.8261	0.8247	0.8237	0.8223	0.8274	0.8244	0.6187	0.6394	0.6476	0.6482	0.9856	1.0000	0.9822	0.9812

Supplementary Tables S3 Clustering performance comparison of DESC with different numbers of highly variable genes measured by NMI and ARI

	NMI						ARI					
Dataset	500	1000	2000	3000	4000	5000	500	1000	2000	3000	4000	5000
Adam	0.7758	0.7807	0.7718	0.7706	0.7756	**0.7842**	0.6499	0.6436	0.5998	0.6082	0.6283	**0.6554**
Bach	0.7201	0.7526	0.7474	**0.7605**	0.7600	0.7550	0.5534	0.6478	0.6098	0.6268	0.6068	**0.6500**
Chen	0.7845	0.8164	**0.8255**	0.8191	0.8127	0.7888	0.5754	0.6960	**0.7312**	0.7178	0.7143	0.6139
Klein	0.6667	0.7121	0.7555	0.7771	**0.8222**	0.8067	0.6168	0.6978	0.7407	0.7651	**0.8084**	0.7659
Muraro	**0.8252**	0.7984	0.7567	0.7483	0.7473	0.7563	**0.7390**	0.6482	0.5249	0.5037	0.5132	0.5239
Plasschaert	0.5979	0.6391	0.6365	**0.6495**	0.6400	0.6437	0.4089	0.4334	0.4554	**0.4687**	0.4497	0.4473
Pollen	**0.9345**	**0.9345**	**0.9345**	0.9326	0.9122	0.9132	0.8508	**0.8512**	0.8508	0.8508	0.8300	0.8307
QS_Diaphragm	0.7691	0.8274	0.8412	**0.8416**	0.8355	0.8413	0.5628	0.6718	0.7058	**0.7232**	0.6765	0.6817
QS_Heart	**0.7553**	0.7340	0.7105	0.7028	0.7059	0.7017	**0.5523**	0.4722	0.4055	0.3807	0.3883	0.3920
QS_Limb_Muscle	0.7696	0.7708	0.8101	**0.8459**	0.8061	0.8220	0.5064	0.4954	0.5930	**0.6638**	0.5806	0.6216
QS_Lung	0.7489	**0.7645**	0.7608	0.7568	0.7427	0.7372	0.4600	0.4675	0.4659	**0.4698**	0.3809	0.3751
QS_Trachea	0.5345	0.5559	0.5527	0.5674	0.5762	**0.5777**	**0.2160**	0.2033	0.1889	0.2026	0.2135	0.2158
Qx_Bladder	**0.5948**	0.5523	0.5579	0.5721	0.5670	0.5654	**0.3350**	0.2689	0.2695	0.3004	0.2946	0.2899
Qx_Limb_Muscle	**0.7735**	0.7513	0.7500	0.7537	0.7630	0.7582	**0.5425**	0.4489	0.4488	0.4521	0.4710	0.4509
Qx_Spleen	0.4427	0.4230	0.4337	0.4355	**0.4457**	0.4444	**0.1979**	0.1284	0.1347	0.1420	0.1486	0.1483
Qx_Trachea	0.4811	**0.4946**	0.4916	0.4831	0.4839	0.4888	0.1343	**0.1442**	0.1351	0.1248	0.1250	0.1313
Romanov	0.5645	0.5974	**0.6020**	0.5798	0.5892	0.5857	0.3421	**0.3873**	0.3864	0.3562	0.3780	0.3663
Tosches_turtle	0.7009	0.7344	0.7994	0.8199	0.8139	**0.8242**	0.6224	0.6759	**0.7562**	0.7542	0.7367	0.7513
Wang_Lung	0.4231	**0.4542**	0.4223	0.4054	0.3846	0.3787	0.2204	**0.2679**	0.2257	0.2044	0.1687	0.1577
Young	0.8005	0.8020	**0.8106**	0.8030	0.7982	0.8016	**0.6945**	0.6818	0.6699	0.6607	0.6565	0.6443

Supplementary Tables S4 Performance comparison of DESC with different numbers of highly variable genes measured by ASW and cLISI

	ASW						cLISI					
Dataset	500	1000	2000	3000	4000	5000	500	1000	2000	3000	4000	5000
Adam	0.6029	0.6031	**0.6109**	0.5865	0.5959	0.5974	0.9542	0.9650	0.9656	**0.9895**	0.9826	0.9864
Bach	0.2286	0.2861	0.3015	**0.3341**	0.3206	0.3263	**1.0000**	**1.0000**	**1.0000**	**1.0000**	**1.0000**	**1.0000**
Chen	0.2731	**0.3709**	0.3514	0.3347	0.3064	0.2913	**0.9998**	0.9997	0.9992	0.9988	0.9990	0.9995
Klein	0.5030	0.5883	0.6370	0.6684	**0.7527**	0.6787	0.9871	0.9971	**1.0000**	**1.0000**	**1.0000**	**1.0000**
Muraro	0.6068	**0.6586**	0.4584	0.5156	0.5134	0.4669	0.9897	0.9988	**0.9990**	0.9986	0.9987	0.9986
Plasschaert	0.2193	0.1982	0.1863	**0.2295**	0.1434	0.1076	**1.0000**	**1.0000**	**1.0000**	**1.0000**	**1.0000**	**1.0000**
Pollen	0.7376	0.7502	0.7767	**0.7780**	0.7472	0.7538	0.8790	0.9547	0.9510	0.9868	0.9645	**0.9898**
QS_Diaphragm	0.8105	0.8290	**0.8556**	0.8484	0.8454	0.8539	**1.0000**	**1.0000**	**1.0000**	**1.0000**	**1.0000**	**1.0000**
QS_Heart	0.5330	0.5307	**0.5674**	0.5334	0.4171	0.4689	**1.0000**	**1.0000**	**1.0000**	**1.0000**	**1.0000**	**1.0000**
QS_Limb_Muscle	0.8412	0.8250	0.8392	**0.8486**	0.8323	0.7824	**1.0000**	**1.0000**	**1.0000**	**1.0000**	**1.0000**	**1.0000**
QS_Lung	**0.4051**	0.3398	0.2914	0.3304	0.3409	0.2878	0.9973	0.9992	0.9996	0.9997	0.9997	**0.9997**
QS_Trachea	**0.4594**	0.4193	0.3645	0.3395	0.4043	0.3530	0.9985	**1.0000**	**1.0000**	**1.0000**	**1.0000**	**1.0000**
Qx_Bladder	**0.6484**	0.5117	0.4761	0.5072	0.4132	0.4117	**1.0000**	**1.0000**	**1.0000**	**1.0000**	**1.0000**	**1.0000**
Qx_Limb_Muscle	**0.7136**	0.6804	0.6518	0.6669	0.6249	0.5610	**1.0000**	**1.0000**	**1.0000**	**1.0000**	**1.0000**	**1.0000**
Qx_Spleen	0.1130	0.1532	0.1221	0.1505	**0.1699**	0.1152	**1.0000**	**1.0000**	**1.0000**	**1.0000**	**1.0000**	**1.0000**
Qx_Trachea	0.1246	**0.1408**	0.1350	0.1257	0.1071	0.1220	**1.0000**	**1.0000**	**1.0000**	**1.0000**	**1.0000**	**1.0000**
Romanov	0.3691	**0.4132**	0.3053	0.2455	0.2239	0.1781	0.9810	0.9863	**0.9887**	0.9848	0.9860	0.9855
Tosches_turtle	0.2274	0.2850	0.3501	**0.4110**	0.3819	0.4044	0.9947	0.9973	0.9987	0.9992	0.9992	**0.9993**
Wang_Lung	0.4521	**0.5860**	0.3716	0.3532	0.2065	0.3185	**1.0000**	**1.0000**	**1.0000**	**1.0000**	**1.0000**	**1.0000**
Young	0.5220	0.5298	0.5325	**0.5351**	0.5163	0.4805	0.9928	0.9949	**0.9959**	0.9924	0.9932	0.9939

Supplementary Tables S5 Performance comparison of DESC with different hidden layer numbers measured by NMI, ARI, ASW and cLISI. DESC is with ([Input layer]-256-32-256-[Output layer]), DESC1 is with ([Input layer]-256-128-32-128-256-[Output layer]), and DESC2 is with ([Input layer]-256-128-64-32-64-128-256-[Output layer])

	NMI			ARI			ASW			cLISI		
Dataset	DESC	DESC1	DESC2	DESC	DESC1	DESC2	DESC	DESC1	DESC2	DESC	DESC1	DESC2
Adam	**0.7706**	0.7671	0.7610	**0.6082**	0.5966	0.5898	**0.5865**	0.5323	0.5297	**0.9895**	0.9857	0.9853
Bach	0.7605	0.7586	**0.7713**	0.6268	0.6629	**0.6928**	**0.3341**	0.2997	0.3212	**1.0000**	**1.0000**	**1.0000**
Chen	0.8191	0.8260	**0.8264**	0.7178	**0.7297**	0.7265	0.3347	**0.3399**	0.3323	0.9988	0.9985	**0.9992**
Klein	**0.7771**	0.7679	0.7575	0.7651	**0.7679**	0.6565	**0.6684**	0.6312	0.6582	**1.0000**	**1.0000**	**1.0000**
Muraro	**0.7483**	0.7437	0.7219	**0.5037**	0.4831	0.4247	0.5156	**0.5205**	0.3642	0.9986	0.9993	**0.9996**
Plasschaert	**0.6495**	0.6114	0.6395	**0.4687**	0.4148	0.4517	**0.2295**	0.1083	0.1881	**1.0000**	**1.0000**	**1.0000**
Pollen	**0.9326**	0.9321	0.9118	**0.8508**	**0.8508**	0.8267	**0.7780**	0.7589	0.7332	0.9868	0.9576	**0.9987**
QS_Diaphragm	**0.8416**	0.7674	0.7214	**0.7232**	0.5697	0.4650	**0.8484**	0.7631	0.6287	**1.0000**	**1.0000**	**1.0000**
QS_Heart	0.7028	0.7084	**0.7107**	0.3807	0.3962	**0.4040**	**0.5334**	0.4269	0.3689	**1.0000**	**1.0000**	**1.0000**
QS_Limb_Muscle	**0.8459**	0.7742	0.7328	**0.6638**	0.5479	0.4522	**0.8486**	0.7361	0.4936	**1.0000**	**1.0000**	**1.0000**
QS_Lung	**0.7568**	0.7441	0.7408	**0.4698**	0.3785	0.3711	**0.3304**	0.1748	0.1639	0.9997	0.9999	**1.0000**
QS_Trachea	**0.5674**	0.5498	0.5484	**0.2026**	0.1988	0.1967	**0.3395**	0.2646	0.2164	**1.0000**	0.9996	0.9999
Qx_Bladder	**0.5721**	0.5103	0.4975	**0.3004**	0.1978	0.1964	**0.5072**	0.2713	0.2030	**1.0000**	**1.0000**	**1.0000**
Qx_Limb_Muscle	**0.7537**	0.7499	0.7459	**0.4521**	0.4477	0.4420	**0.6669**	0.6073	0.5862	**1.0000**	**1.0000**	**1.0000**
Qx_Spleen	0.4355	0.4340	**0.4494**	0.1420	0.1708	**0.1746**	**0.1505**	0.0463	0.0835	**1.0000**	**1.0000**	**1.0000**
Qx_Trachea	0.4831	0.4906	**0.4914**	0.1248	0.1363	**0.1368**	**0.1257**	0.0586	0.0857	**1.0000**	**1.0000**	**1.0000**
Romanov	**0.5798**	0.5620	0.5483	**0.3562**	0.3512	0.3478	**0.2455**	0.1826	0.2348	**0.9848**	0.9730	0.9714
Tosches_turtle	**0.8199**	0.7804	0.7555	**0.7542**	0.6817	0.7201	**0.4110**	0.2806	0.2815	**0.9992**	0.9986	0.9979
Wang_Lung	0.4054	**0.4352**	0.4268	0.2044	**0.2465**	0.2351	0.3532	**0.4065**	0.3542	**1.0000**	**1.0000**	**1.0000**
Young	0.8030	**0.8055**	0.8021	0.6607	**0.6674**	0.6432	**0.5351**	0.5087	0.5018	0.9924	0.9960	**0.9983**

Supplementary Tables S6 Clustering performance comparison of scDeepCluster with different numbers of highly variable genes measured by NMI and ARI

	NMI						ARI					
Dataset	500	1000	2000	3000	4000	5000	500	1000	2000	3000	4000	5000
Adam	0.7302	0.7686	**0.7788**	0.7754	0.7770	0.7619	0.5800	0.6698	0.6888	0.6911	0.6952	**0.7129**
Bach	**0.8092**	0.7895	0.7580	0.7572	0.7590	0.7551	**0.8029**	0.6520	0.6129	0.6106	0.6113	0.6072
Chen	**0.7465**	0.7422	0.7178	0.6951	0.6798	0.6613	0.5163	**0.5221**	0.3893	0.3286	0.3007	0.2714
Klein	**0.7767**	0.7582	0.7619	0.6450	0.6379	0.6245	**0.8132**	0.7317	0.7170	0.5266	0.5125	0.5065
Muraro	**0.8167**	0.8159	0.7795	0.7798	0.7703	0.7669	**0.7866**	0.6976	0.6248	0.6154	0.6149	0.6033
Plasschaert	**0.6310**	0.6158	0.6207	0.6132	0.6148	0.5795	**0.4791**	0.4421	0.4352	0.4172	0.4201	0.3672
Pollen	0.9341	**0.9380**	0.9151	0.9199	0.9225	0.9174	0.9389	**0.9425**	0.8437	0.8439	0.8466	0.8427
QS_Diaphragm	0.9297	**0.9534**	0.9481	0.9294	0.8022	0.8037	0.9548	**0.9760**	0.9749	0.9650	0.6584	0.6596
QS_Heart	**0.8613**	0.8193	0.8038	0.7695	0.7628	0.7335	**0.9108**	0.6704	0.6580	0.5888	0.5923	0.5355
QS_Limb_Muscle	0.9510	**0.9511**	0.8186	0.8143	0.8153	0.8099	**0.9730**	0.9716	0.6661	0.6626	0.6584	0.6578
QS_Lung	**0.7477**	0.7297	0.7229	0.7298	0.7221	0.7107	**0.6000**	0.4944	0.4803	0.4905	0.4813	0.4043
QS_Trachea	**0.7604**	0.6511	0.6679	0.6442	0.6375	0.6420	**0.8298**	0.5441	0.5482	0.5153	0.4967	0.5046
Qx_Bladder	**0.7805**	0.7788	0.7565	0.7543	0.7550	0.7640	0.7357	**0.7381**	0.7314	0.7270	0.7302	0.7351
Qx_Limb_Muscle	0.9746	0.9743	**0.9746**	0.9707	0.9603	0.9450	**0.9875**	0.9844	0.9861	0.9848	0.9768	0.9583
Qx_Spleen	**0.8102**	0.6039	0.5172	0.5170	0.5384	0.4869	**0.9005**	0.4924	0.3070	0.3424	0.3228	0.3121
Qx_Trachea	**0.6525**	0.5320	0.4466	0.3960	0.3712	0.3734	**0.5069**	0.3115	0.2107	0.1394	0.1480	0.1438
Romanov	0.6812	**0.6958**	0.6904	0.6684	0.6308	0.5919	**0.6715**	0.6680	0.6512	0.6114	0.5377	0.5332
Tosches_turtle	0.6836	**0.7160**	0.7090	0.7141	0.7035	0.6954	0.3907	0.4175	0.4213	**0.4649**	0.4562	0.4475
Wang_Lung	0.8870	0.8974	**0.9024**	0.8517	0.6647	0.4547	0.9462	**0.9522**	0.9515	0.9173	0.7245	0.4707
Young	**0.7550**	0.7505	0.7332	0.6726	0.6430	0.6185	**0.6292**	0.6197	0.6182	0.5328	0.5023	0.4681

Supplementary Tables S7 Comparison performance of scDeepCluster with different numbers of highly variable genes measured by ASW and cLISI

	ASW						cLISI					
Dataset	500	1000	2000	3000	4000	5000	500	1000	2000	3000	4000	5000
Adam	0.5736	0.5994	**0.6009**	0.5982	0.5916	0.5860	**0.9457**	0.9395	0.9337	0.9139	0.8828	0.8572
Bach	**0.5156**	0.5075	0.4835	0.4550	0.4473	0.4464	0.9916	**1.0000**	0.9992	0.9993	0.9981	0.9988
Chen	0.4330	**0.4744**	0.4712	0.4441	0.4145	0.4012	**0.9902**	0.9737	0.9729	0.9723	0.9663	0.9676
Klein	0.6347	0.6876	**0.6988**	0.6865	0.6600	0.6803	0.9960	0.9974	0.9969	0.9960	0.9969	**0.9975**
Muraro	0.4924	**0.6526**	0.5737	0.5933	0.5906	0.5924	0.9806	**0.9923**	0.9740	0.9361	0.9128	0.9205
Plasschaert	0.3690	0.4094	0.4377	0.4499	0.4547	**0.4881**	**1.0000**	0.9998	0.9998	0.9995	0.9995	0.9989
Pollen	0.7674	0.7640	0.7560	0.7521	**0.7954**	0.7420	0.9268	**0.9286**	0.8482	0.8787	0.8930	0.8720
QS_Diaphragm	0.7758	**0.8412**	0.8187	0.8130	0.7791	0.7674	**1.0000**	**1.0000**	**1.0000**	**1.0000**	**1.0000**	**1.0000**
QS_Heart	0.6467	**0.6856**	0.6822	0.6729	0.6714	0.6587	**1.0000**	**1.0000**	**1.0000**	**1.0000**	0.9999	0.9996
QS_Limb_Muscle	0.7638	0.7881	0.7968	0.7833	**0.8033**	0.7730	**1.0000**	**1.0000**	**1.0000**	**1.0000**	**1.0000**	**1.0000**
QS_Lung	0.4725	0.4102	0.5227	0.4769	0.5240	**0.5356**	0.9960	**0.9986**	0.9973	0.9985	0.9954	0.9965
QS_Trachea	0.5669	0.5518	0.5765	0.5552	**0.6005**	0.5861	0.9899	0.9932	**0.9976**	0.9942	0.9928	0.9911
Qx_Bladder	**0.6674**	0.6107	0.5989	0.5229	0.4515	0.3679	**1.0000**	**1.0000**	**1.0000**	**1.0000**	**1.0000**	**1.0000**
Qx_Limb_Muscle	0.7866	0.7827	**0.7908**	0.7884	0.7833	0.7768	**1.0000**	**1.0000**	**1.0000**	**1.0000**	**1.0000**	0.9992
Qx_Spleen	**0.6116**	0.5893	0.5007	0.5204	0.4937	0.4937	**1.0000**	**1.0000**	**1.0000**	**1.0000**	**1.0000**	**1.0000**
Qx_Trachea	0.4464	0.4507	0.4615	**0.4632**	0.4422	0.4515	**1.0000**	0.9945	0.9913	0.9832	0.9617	0.9510
Romanov	0.3462	0.3718	**0.3863**	0.3537	0.3403	0.3086	0.9371	**0.9895**	0.9856	0.9847	0.9754	0.9668
Tosches_turtle	0.3943	0.4606	0.4961	**0.5235**	0.5197	0.5021	**0.9538**	0.9536	0.9428	0.9375	0.9297	0.9303
Wang_Lung	**0.6232**	0.5052	0.4380	0.4051	0.3939	0.4060	**1.0000**	**1.0000**	**1.0000**	**1.0000**	**1.0000**	**1.0000**
Young	0.4217	**0.4514**	0.4136	0.3918	0.3578	0.3420	**0.9265**	0.9216	0.9116	0.9063	0.9026	0.9006

Supplementary Tables S8 Comparison performance of scDeepCluster with different hidden layer numbers measured by NMI, ARI, ASW and cLISI. scDeepCluster is with ([Input layer]-256-32-256-[Output layer]), scDeepCluster1 is with ([Input layer]-256-128-32-128-256-[Output layer]), and scDeepCluster2 is with ([Input layer]-256-128-64-32-64-128-256-[Output layer])

	NMI			ARI			ASW			cLISI		
Dataset	scDeepCluster	scDeepCluster1	scDeepCluster2	scDeepCluster	scDeepCluster1	scDeepCluster2	scDeepCluster	scDeepCluster1	scDeepCluster2	scDeepCluster	scDeepCluster1	scDeepCluster2
Adam	0.73015	0.69389	0.725605	0.579965	0.507285	0.597535	0.5736	0.55335	0.5743	0.94574	0.900385	0.916535
Bach	0.809215	0.74128	0.734875	0.802885	0.60128	0.591655	0.51565	0.40935	0.40595	0.991625	0.977985	0.96298
Chen	**0.7465**	0.7226	0.7198	**0.5163**	0.4268	0.4431	**0.4330**	0.3674	0.3464	**0.9902**	0.9893	0.9899
Klein	**0.7767**	0.7606	0.7052	**0.8132**	0.7486	0.6812	0.6347	**0.6855**	0.6419	0.9960	**0.9973**	0.9963
Muraro	0.8167	0.8366	**0.8607**	0.7866	0.8032	**0.9130**	0.4924	0.4833	**0.5781**	0.9806	**0.9904**	0.9892
Plasschaert	0.6310	**0.6652**	0.6629	0.4791	0.5596	**0.6066**	0.3690	**0.4218**	0.3596	**1.0000**	0.9992	0.9978
Pollen	0.9341	0.9033	**0.9369**	0.9389	0.8555	**0.9422**	**0.7674**	0.6634	0.7381	**0.9268**	0.8780	0.9138
QS_Diaphragm	0.9297	**0.9343**	0.9250	0.9548	**0.9665**	0.9636	**0.7758**	0.7353	0.7097	**1.0000**	0.9969	0.9988
QS_Heart	0.8613	**0.8705**	0.7969	0.9108	**0.9252**	0.6685	**0.6467**	0.6004	0.5743	**1.0000**	0.9980	0.9963
QS_Limb_Muscle	**0.9510**	0.8726	0.9209	**0.9730**	0.9091	0.9616	**0.7638**	0.7134	0.7099	**1.0000**	0.9994	0.9995
QS_Lung	**0.7477**	0.7126	0.7296	**0.6000**	0.4850	0.5131	**0.4725**	0.4242	0.4703	**0.9960**	0.9829	0.9775
QS_Trachea	**0.7604**	0.5714	0.5331	**0.8298**	0.5219	0.5177	**0.5669**	0.4901	0.5259	**0.9899**	0.9584	0.9707
Qx_Bladder	**0.7805**	0.7626	0.7790	0.7357	0.7313	**0.7483**	**0.6674**	0.4437	0.4794	**1.0000**	0.9999	**1.0000**
Qx_Limb_Muscle	**0.9746**	0.9413	0.9116	**0.9875**	0.9609	0.9397	**0.7866**	0.7516	0.7407	**1.0000**	0.9987	0.9968
Qx_Spleen	**0.8102**	0.6067	0.5778	**0.9005**	0.4918	0.4945	**0.6116**	0.4816	0.4580	**1.0000**	0.9993	0.9981
Qx_Trachea	**0.6525**	0.3666	0.3988	**0.5069**	0.1211	0.1848	**0.4464**	0.3947	0.3909	**1.0000**	0.9545	0.9344
Romanov	**0.6812**	0.6564	0.6408	**0.6715**	0.6445	0.6244	**0.3462**	0.2874	0.3141	0.9371	**0.9713**	0.9626
Tosches_turtle	0.6836	**0.7407**	0.7230	0.3907	**0.5445**	0.5188	0.3943	**0.4614**	0.4452	0.9538	0.9536	**0.9584**
Wang_Lung	**0.8870**	0.2317	0.2337	**0.9462**	0.0673	0.0867	**0.6232**	0.4298	0.4265	**1.0000**	**1.0000**	**1.0000**
Young	**0.7550**	0.7270	0.7122	**0.6292**	0.6208	0.6061	0.4217	0.4031	**0.4337**	0.9265	**0.9430**	0.9406

Supplementary Tables S9 Clustering performance comparison of DCA with different numbers of highly variable genes measured by NMI and ARI

			NMI						ARI			heightDataset
500	1000	2000	3000	4000	5000	500	1000	2000	3000	4000	5000	
Adam	0.7706	0.7357	**0.7947**	0.7314	0.7256	0.7191	0.7193	0.6260	**0.7723**	0.6287	0.6352	0.6308
Bach	**0.8340**	0.7970	0.7952	0.8004	0.8015	0.7911	**0.8364**	0.6550	0.6568	0.6619	0.6568	0.6526
Chen	0.6991	**0.7002**	0.6841	0.6751	0.6727	0.6667	**0.3524**	0.3059	0.3181	0.3020	0.3179	0.3081
Klein	0.7062	**0.7435**	0.5911	0.4854	0.5294	0.5653	0.6996	**0.7735**	0.4801	0.3913	0.4313	0.4483
Muraro	0.7650	**0.8345**	0.8252	0.8247	0.8311	0.7733	0.6477	**0.8800**	0.6970	0.7012	0.7112	0.6509
Plasschaert	**0.6709**	0.6521	0.6294	0.6307	0.6177	0.6365	**0.5668**	0.4722	0.4191	0.4478	0.4486	0.4744
Pollen	0.9002	0.9212	0.9161	**0.9513**	0.9506	0.9476	0.8409	0.8755	0.8411	0.8834	**0.8937**	0.8822
QS_Diaphragm	0.9243	0.9424	**0.9461**	0.8663	0.7980	0.8179	0.9602	0.9704	**0.9717**	0.8899	0.6569	0.6724
QS_Heart	0.7878	**0.8164**	0.8132	0.8044	0.7812	0.8109	0.6534	**0.6719**	0.6684	0.6588	0.6273	0.6676
QS_Limb_Muscle	0.9231	0.8700	**0.9453**	0.8161	0.8174	0.8228	0.9599	0.8098	**0.9671**	0.6633	0.6608	0.6752
QS_Lung	0.7602	**0.7666**	0.7292	0.7267	0.7480	0.7335	**0.5957**	0.5903	0.4882	0.4982	0.5357	0.4979
QS_Trachea	0.7041	0.7439	0.6684	0.6476	**0.7474**	0.5933	0.5503	0.8296	0.5925	0.4884	**0.8304**	0.4833
Qx_Bladder	0.7736	0.7812	0.7939	0.7694	0.7782	**0.8064**	0.7451	0.7460	0.7532	0.7398	0.7442	**0.7569**
Qx_Limb_Muscle	**0.9241**	0.9068	0.8517	0.8562	0.8379	0.8439	**0.9539**	0.9311	0.7935	0.7924	0.7890	0.7819
Qx_Spleen	0.7698	**0.7938**	0.6379	0.6187	0.6274	0.6234	0.8819	**0.8984**	0.4956	0.4660	0.4858	0.4745
Qx_Trachea	0.7041	0.7093	0.7068	0.7150	**0.7155**	0.6993	0.5503	0.5476	0.5304	**0.5522**	0.5521	0.5288
Romanov	**0.6951**	0.5966	0.5924	0.6150	0.5250	0.5174	**0.7295**	0.5778	0.5643	0.5686	0.4352	0.4461
Tosches_turtle	0.6593	0.6534	**0.6707**	0.6388	0.6386	0.6262	**0.4573**	0.3819	0.4301	0.4005	0.4285	0.4363
Wang_Lung	0.9207	**0.9352**	0.9299	0.9139	0.9223	0.9235	0.9678	**0.9745**	0.9722	0.9640	0.9685	0.9692
Young	0.7514	**0.7619**	0.7186	0.6549	0.6402	0.6346	0.6319	**0.6562**	0.5404	0.4494	0.4191	0.4247

Supplementary Tables S10 Performance comparison of DCA with different numbers of highly variable genes measured by ASW and cLISI

	ASW						cLISI					
Dataset	500	1000	2000	3000	4000	5000	500	1000	2000	3000	4000	5000
Adam	**0.5841**	0.5741	0.5713	0.5600	0.5501	0.5395	0.8074	0.8617	0.8760	**0.9179**	0.8603	0.8744
Bach	**0.5867**	0.5833	0.5185	0.5381	0.5213	0.4994	0.9858	**0.9999**	0.9964	0.9998	0.9991	0.9918
Chen	0.4955	**0.5041**	0.4476	0.4333	0.4462	0.3867	0.9816	0.9942	0.9923	**0.9955**	0.9851	0.9893
Klein	0.6663	0.6842	**0.6900**	0.6389	0.6123	0.5859	0.9892	0.9894	**0.9952**	0.9942	0.9006	0.8017
Muraro	0.4465	**0.7564**	0.6493	0.6407	0.6514	0.3757	0.9582	0.9484	**0.9935**	0.9871	0.9485	0.9640
Plasschaert	0.4148	0.4688	0.4138	**0.4829**	0.3889	0.4230	**1.0000**	**1.0000**	**1.0000**	**1.0000**	0.9987	0.9995
Pollen	0.7616	0.7280	0.8004	**0.8071**	0.7900	0.7966	0.8863	0.8691	**1.0000**	0.9661	0.8996	0.9436
QS_Diaphragm	0.7833	0.8366	0.8076	**0.8674**	0.8054	0.7978	0.9777	0.9990	0.9992	**1.0000**	**1.0000**	**1.0000**
QS_Heart	0.6991	**0.7618**	0.7231	0.7000	0.7019	0.6751	0.9966	**1.0000**	**1.0000**	0.9938	**1.0000**	**1.0000**
QS_Limb_Muscle	0.8076	0.8201	**0.8266**	0.8257	0.8026	0.8150	0.9996	0.9996	**1.0000**	**1.0000**	**1.0000**	**1.0000**
QS_Lung	0.5843	0.5525	0.5554	0.5295	0.5593	**0.5914**	0.9883	0.9858	0.9972	0.9829	0.9958	**0.9989**
QS_Trachea	0.5813	0.6038	0.6011	**0.6703**	0.6596	0.6663	**1.0000**	0.9799	0.9915	0.9941	0.9981	0.9989
Qx_Bladder	**0.7488**	0.6884	0.7014	0.7486	0.7125	0.6638	**1.0000**	**1.0000**	**1.0000**	**1.0000**	**1.0000**	**1.0000**
Qx_Limb_Muscle	**0.8324**	0.8223	0.7879	0.8035	0.7899	0.7772	0.9953	0.9992	**1.0000**	0.9995	**1.0000**	0.9963
Qx_Spleen	**0.7241**	0.7096	0.6403	0.6166	0.6008	0.5614	**1.0000**	**1.0000**	**1.0000**	**1.0000**	**1.0000**	**1.0000**
Qx_Trachea	**0.5813**	0.5101	0.4815	0.4639	0.4989	0.4859	**1.0000**	**1.0000**	**1.0000**	**1.0000**	**1.0000**	**1.0000**
Romanov	0.3551	0.3499	**0.4047**	0.3988	0.3963	0.3908	0.9176	0.8788	**0.9634**	0.9623	0.9363	0.9214
Tosches_turtle	0.4442	0.4439	**0.4521**	0.4356	0.4029	0.3994	0.9359	**0.9567**	0.9388	0.9379	0.9500	0.9158
Wang_Lung	**0.6649**	0.5846	0.4323	0.4160	0.4113	0.3642	**1.0000**	**1.0000**	**1.0000**	**1.0000**	**1.0000**	**1.0000**
Young	0.3945	0.4145	**0.4266**	0.3899	0.3595	0.3416	0.9063	0.9058	**0.9165**	0.8964	0.8875	0.8914

Supplementary Tables S11 Performance comparison of DCA with different hidden layer numbers measured by NMI, ARI, ASW and cLISI. DCA is with ([Input layer]-256-32-256-[Output layer]), DAC1 is with ([Input layer]-256-128-32-128-256-[Output layer]), and DCA2 is with ([Input layer]-256-128-64-32-64-128-256-[Output layer])

	NMI			ARI			ASW			cLISI		
Dataset	DCA	DCA1	DCA2	DCA	DCA1	DCA2	DCA	DCA1	DCA2	DCA	DCA1	DCA2
Adam	**0.7357**	0.7013	0.7103	**0.6260**	0.5987	0.6165	**0.5741**	0.5415	0.5298	0.8617	0.8472	**0.8703**
Bach	**0.7970**	0.7605	0.7406	**0.6550**	0.6050	0.5774	**0.5833**	0.5012	0.4440	**0.9999**	0.9995	0.9998
Chen	**0.7002**	0.6757	0.6580	0.3059	**0.3159**	0.2904	**0.5041**	0.4487	0.3967	**0.9942**	0.9861	0.9852
Klein	**0.7435**	0.5429	0.4694	**0.7735**	0.4284	0.4000	**0.6842**	0.5554	0.5093	**0.9894**	0.8522	0.9010
Muraro	**0.8345**	0.8006	0.7590	**0.8800**	0.6859	0.6205	**0.7564**	0.6314	0.5907	0.9484	0.9731	**0.9802**
Plasschaert	**0.6521**	0.6267	0.5726	0.4722	**0.4825**	0.3974	**0.4688**	0.4499	0.3592	**1.0000**	0.9991	0.9996
Pollen	**0.9212**	0.9204	0.9143	**0.8755**	0.8503	0.8372	0.7280	**0.8001**	0.7729	0.8691	**0.9217**	0.8958
QS_Diaphragm	0.9424	**0.9450**	0.9228	0.9704	**0.9741**	0.9605	**0.8366**	0.8105	0.7927	0.9990	**0.9995**	0.9953
QS_Heart	0.8164	**0.8421**	0.8219	0.6719	**0.8694**	0.8493	**0.7618**	0.7348	0.6598	**1.0000**	0.9976	**1.0000**
QS_Limb_Muscle	0.8700	0.8325	**0.8823**	0.8098	0.6749	**0.9323**	0.8201	**0.8270**	0.7908	0.9996	**1.0000**	0.9993
QS_Lung	**0.7666**	0.7444	0.7601	0.5903	0.5739	**0.6497**	0.5525	**0.5907**	0.5546	0.9858	**0.9914**	0.9832
QS_Trachea	**0.7439**	0.5977	0.6381	**0.8296**	0.5400	0.5605	0.6038	**0.6348**	0.5928	0.9799	0.9856	**0.9949**
Qx_Bladder	0.7812	**0.7911**	0.7753	0.7460	**0.7515**	0.7489	0.6884	**0.7568**	0.7257	**1.0000**	**1.0000**	**1.0000**
Qx_Limb_Muscle	**0.9068**	0.8288	0.8255	**0.9311**	0.6897	0.7536	**0.8223**	0.8026	0.7856	**0.9992**	0.9988	0.9984
Qx_Spleen	**0.7938**	0.6240	0.5836	**0.8984**	0.4855	0.4831	**0.7096**	0.6732	0.5713	**1.0000**	**1.0000**	**1.0000**
Qx_Trachea	**0.7093**	0.7019	0.5226	**0.5476**	0.5402	0.3183	**0.5101**	0.4784	0.4186	**1.0000**	**1.0000**	**1.0000**
Romanov	**0.5966**	0.5776	0.5391	**0.5778**	0.5511	0.5364	0.3499	0.3748	**0.3760**	0.8788	**0.9392**	0.9376
Tosches_turtle	**0.6534**	0.6257	0.5894	0.3819	**0.4072**	0.3533	**0.4439**	0.4422	0.3630	**0.9567**	0.9433	0.9515
Wang_Lung	**0.9352**	0.9256	0.8910	**0.9745**	0.9694	0.9524	**0.5846**	0.4532	0.3955	**1.0000**	**1.0000**	**1.0000**
Young	**0.7619**	0.6769	0.6033	**0.6562**	0.4702	0.3774	**0.4145**	0.3794	0.3801	**0.9058**	0.8961	0.8974

Supplementary Fig. 12 Performance of scBGEDA and other deep learning-based models with BGEC integrated measured by NMI and ARI

	NMI					ARI				
Dataset	scBGEDA	DESC	DCA	scziDesk	scDeepCluster	scBGEDA	DESC	DCA	scziDesk	scDeepCluster
Adam	0.8938	0.8780	0.7629	**0.8956**	0.7590	0.9061	0.8843	0.7315	**0.9130**	0.6734
Bach	**0.8307**	0.7594	0.8183	0.7972	0.7474	**0.8453**	0.7927	0.7839	0.8284	0.6603
Chen	0.6410	0.6778	0.8627	0.5648	**0.9255**	0.3590	0.4810	0.7134	0.2824	**0.8674**
Klein	0.8471	0.7699	0.5444	**0.8783**	0.7169	0.8688	0.7513	0.4527	**0.9118**	0.6835
Muraro	**0.8817**	0.7779	0.7619	0.8175	0.8513	**0.9271**	0.7352	0.6607	0.7219	0.7708
Plasschaert	0.5922	0.5242	0.7345	**0.8079**	0.6734	0.5957	0.5024	0.6580	**0.8855**	0.6874
Pollen	0.9231	0.9035	**0.9660**	0.9023	0.9535	0.8759	0.8459	**0.9659**	0.8887	0.9601
QS_Diaphragm	**0.9611**	0.9577	0.9512	0.5991	0.8954	**0.9830**	0.9813	0.9760	0.4835	0.9540
QS_Heart	**0.9166**	0.7246	0.7927	0.9110	0.7652	**0.9647**	0.6656	0.8723	0.9540	0.6970
QS_Limb_Muscle	0.9609	**0.9647**	0.8929	0.6577	0.9197	0.9801	**0.9809**	0.9480	0.5518	0.9565
QS_Lung	0.7884	0.6769	0.8365	0.8323	**0.8445**	0.7660	0.5247	**0.8284**	0.7495	0.8095
QS_Trachea	**0.8929**	0.6226	0.6394	0.8372	0.5578	**0.9309**	0.5316	0.5931	0.8838	0.4978
Qx_Bladder	**0.9863**	0.7118	0.6965	0.9800	0.6943	**0.9960**	0.7588	0.7508	0.9932	0.7546
Qx_Limb_Muscle	0.9864	**0.9872**	0.8217	0.9342	0.8973	0.9941	**0.9943**	0.7665	0.9599	0.9282
Qx_Spleen	0.8389	0.4315	0.5749	**0.8774**	0.5614	0.9221	0.3240	0.5280	**0.9466**	0.5460
Qx_Trachea	0.6414	0.5405	0.6880	**0.8170**	0.5325	0.5546	0.4168	0.6534	**0.9115**	0.3919
Romanov	0.7388	0.6529	0.6161	**0.7816**	0.6855	0.8059	0.6713	0.5837	**0.8131**	0.6766
Tosches_turtle	0.7510	0.7622	0.7647	0.5306	**0.7673**	0.7383	**0.7633**	0.6383	0.3947	0.6085
Wang_Lung	0.6151	**0.9189**	0.6371	0.9131	0.4504	0.7323	**0.9676**	0.7512	0.9647	0.4552
Young	0.7759	**0.8404**	0.7203	0.8025	0.7854	0.6996	**0.8113**	0.5728	0.7696	0.7087
Average	**0.8232**	0.7541	0.7541	0.8069	0.7492	**0.8223**	0.7192	0.7214	0.7904	0.7144

Supplementary Fig. 13 Performance of scBGEDA and other deep learning-based models with BGEC integrated measured by ASW and cLISI

	ASW					cLISI				
Dataset	scBGEDA	DESC	DCA	scziDesk	scDeepCluster	scBGEDA	DESC	DCA	scziDesk	scDeepCluster
Adam	**0.6298**	0.6109	0.5298	0.6111	0.5743	**1.0000**	0.9656	0.8703	0.8967	0.9165
Bach	0.5661	0.3015	0.4440	**0.6193**	0.4060	**1.0000**	**1.0000**	0.9998	0.9943	0.9630
Chen	**0.5897**	0.3514	0.3967	0.5091	0.3464	**1.0000**	0.9992	0.9852	0.9802	0.9899
Klein	**0.7265**	0.6370	0.5093	0.5901	0.6419	**1.0000**	**1.0000**	0.9010	0.9919	0.9963
Muraro	**0.5933**	0.4584	0.5907	0.5202	0.5781	**1.0000**	0.9990	0.9802	0.8706	0.9892
Plasschaert	**0.4536**	0.1863	0.3592	0.4224	0.3596	**1.0000**	**1.0000**	0.9996	0.9982	0.9978
Pollen	**0.7783**	0.7767	0.7729	0.7362	0.7381	**1.0000**	0.9510	0.8958	0.9065	0.9138
QS_Diaphragm	**0.8772**	0.8556	0.7927	0.8487	0.7097	**1.0000**	**1.0000**	0.9953	0.9938	0.9988
QS_Heart	**0.7176**	0.5674	0.6598	0.7129	0.5743	**1.0000**	**1.0000**	**1.0000**	0.9881	0.9963
QS_Limb_Muscle	**0.8524**	0.8392	0.7908	0.8360	0.7099	**1.0000**	**1.0000**	0.9993	0.9970	0.9995
QS_Lung	**0.6289**	0.2914	0.5546	0.5448	0.4703	**1.0000**	0.9996	0.9832	0.9842	0.9775
QS_Trachea	**0.6655**	0.3645	0.5928	0.5550	0.5259	**1.0000**	**1.0000**	0.9949	0.9853	0.9707
Qx_Bladder	0.7609	0.4761	0.7257	**0.7856**	0.4794	**1.0000**	**1.0000**	**1.0000**	**1.0000**	**1.0000**
Qx_Limb_Muscle	0.8262	0.6518	0.7856	**0.8274**	0.7407	**1.0000**	**1.0000**	0.9984	0.9937	0.9968
Qx_Spleen	0.6772	0.1221	0.5713	**0.7273**	0.4580	**1.0000**	**1.0000**	**1.0000**	**1.0000**	0.9981
Qx_Trachea	0.5018	0.1350	0.4186	**0.5967**	0.3909	**1.0000**	**1.0000**	**1.0000**	0.9991	0.9344
Romanov	**0.4517**	0.3053	0.3760	0.3831	0.3141	**1.0000**	0.9887	0.9376	0.8877	0.9626
Tosches_turtle	**0.5998**	0.3501	0.3630	0.4852	0.4452	**1.0000**	0.9987	0.9515	0.9300	0.9584
Wang_Lung	0.4380	0.3716	0.3955	**0.6947**	0.4265	**1.0000**	**1.0000**	**1.0000**	**1.0000**	**1.0000**
Young	0.4526	**0.5325**	0.3801	0.4159	0.4337	**1.0000**	0.9959	0.8974	0.9196	0.9406
Average	**0.6394**	0.4592	0.5504	0.6211	0.5161	**1.0000**	0.9949	0.9695	0.9658	0.9750

Supplementary Table S14: Clustering performance comparison of the different clustering algorithms on twenty real scRNA-seq datasets measured by NMI

Algorithm	scBGEDA	scziDesk	SHARP	DESC	scDeepCluster	CIDR	SOUP	SC3	Scanpy	Seurat	DCA	PCA
Adam	**0.8938**	0.8609	0.1444	0.7706	0.7302	0.0510	0.4768	0.2068	0.4794	0.6217	0.7357	0.0218
Bach	0.8307	**0.8812**	0.4603	0.7605	0.8092	0.8194	0.4790	0.5330	0.3866	0.5048	0.7970	0.4807
Chen	0.6410	0.7771	0.5755	**0.8191**	0.7465	0.1685	0.1399	0.2409	0.5079	0.6532	0.7002	0.2655
Klein	**0.8471**	0.7874	0.4944	0.7771	0.7767	0.7308	0.4712	0.3091	0.3597	0.5253	0.7435	0.2979
Muraro	**0.8817**	0.7722	0.3063	0.7483	0.8167	0.5274	0.5336	0.3254	0.4252	0.5117	0.8345	0.4953
Plasschaert	0.5922	0.8103	**0.8732**	0.6495	0.6310	0.3848	0.8063	0.3383	0.2706	0.2898	0.6521	0.3072
Pollen	0.9231	0.8873	0.0903	0.9326	**0.9341**	0.8224	0.4152	0.8866	0.8511	0.7960	0.9212	0.8565
QS_Diaphragm	0.9611	0.7071	0.9443	0.8416	0.9297	**0.9693**	0.4615	0.6981	0.4809	0.4499	0.9424	0.1617
QS_Heart	**0.9166**	0.8836	0.6888	0.7028	0.8613	0.6155	0.7793	0.4466	0.2921	0.3194	0.8164	0.1165
QS_Limb_Muscle	**0.9609**	0.7580	0.9350	0.8459	0.9510	0.7811	0.7923	0.5649	0.5072	0.4652	0.8700	0.0354
QS_Lung	0.7884	0.7648	**0.8124**	0.7568	0.7477	0.3162	0.2105	0.4214	0.3009	0.3204	0.7666	0.1439
QS_Trachea	**0.8929**	0.6842	0.7416	0.5674	0.7604	0.5387	0.3932	0.5191	0.1915	0.2828	0.7439	-0.0041
Qx_Bladder	**0.9863**	0.9734	0.8933	0.5721	0.7805	0.7341	0.9407	0.5168	0.1893	0.2369	0.7812	0.5375
Qx_Limb_Muscle	**0.9864**	0.9138	0.8044	0.7537	0.9746	0.6767	0.5320	0.7239	0.4193	0.4765	0.9068	0.3778
Qx_Spleen	0.8389	0.8640	0.6541	0.4355	0.8102	0.6484	**0.9055**	0.2486	0.1426	0.1547	0.7938	0.0766
Qx_Trachea	0.6414	**0.8384**	0.5094	0.4831	0.6525	0.3401	0.2695	0.1334	0.1134	0.1476	0.7093	-0.0644
Romanov	**0.7388**	0.7300	0.5917	0.5798	0.6812	0.3223	0.3258	0.2955	0.2546	0.3041	0.5966	0.1270
Tosches_turtle	0.7510	0.7281	0.5221	**0.8199**	0.6836	0.2586	0.1073	0.3266	0.4399	0.5133	0.6534	0.4114
Wang_Lung	0.6151	0.8222	0.9256	0.4054	0.8870	0.3925	**0.9673**	0.9338	0.1420	0.1212	0.9352	0.9245
Young	0.7759	0.7876	0.2547	**0.8030**	0.7550	0.2656	0.5024	0.2393	0.3141	0.5207	0.7619	0.2047
Average	**0.8232**	0.8116	0.6472	0.7012	0.7960	0.6030	0.6449	0.5809	0.6355	0.6613	0.7831	0.3695

Supplementary Table S15: Clustering performance comparison of the different clustering algorithms on twenty real scRNA-seq datasets measured by ARI

Algorithm	scBGEDA	scziDesk	SHARP	DESC	scDeepCluster	CIDR	SOUP	SC3	Scanpy	Seurat	DCA	PCA
Adam	**0.9061**	0.8726	0.3262	0.6082	0.5800	0.1231	0.6610	0.3370	0.6924	0.7717	0.6260	0.0720
Bach	0.8453	**0.8981**	0.5473	0.6268	0.8029	0.7872	0.6644	0.6499	0.6966	0.7345	0.6550	0.4913
Chen	0.3590	**0.7896**	0.6434	0.7178	0.5163	0.3966	0.3752	0.5875	0.7256	0.7309	0.3059	0.5212
Klein	**0.8688**	0.8023	0.6465	0.7651	0.8132	0.7079	0.6230	0.4315	0.6608	0.7382	0.7735	0.4150
Muraro	**0.9271**	0.6832	0.4595	0.5037	0.7866	0.6522	0.6307	0.5288	0.7189	0.7490	0.8800	0.7024
Plasschaert	0.5957	**0.8722**	0.7995	0.4687	0.4791	0.5752	0.7434	0.5695	0.5600	0.5855	0.4722	0.3964
Pollen	0.8759	0.8723	0.3288	0.8508	**0.9389**	0.9131	0.6778	0.9353	0.9294	0.8864	0.8755	0.9235
QS_Diaphragm	**0.9830**	0.4759	0.9005	0.7232	0.9548	0.9250	0.6593	0.8013	0.7394	0.7391	0.9704	0.2537
QS_Heart	**0.9647**	0.9255	0.6944	0.3807	0.9108	0.7418	0.8004	0.6527	0.6525	0.6808	0.6719	0.2097
QS_Limb_Muscle	**0.9801**	0.5438	0.8844	0.6638	0.9730	0.8401	0.7481	0.7175	0.7514	0.7525	0.8098	0.0937
QS_Lung	0.7660	0.6128	**0.7709**	0.4698	0.6000	0.5628	0.5180	0.6730	0.7052	0.6861	0.5903	0.2314
QS_Trachea	**0.9309**	0.7428	0.7588	0.2026	0.8298	0.6103	0.4762	0.5687	0.5425	0.6031	0.8296	0.1102
Qx_Bladder	**0.9960**	0.9869	0.8604	0.3004	0.7357	0.7732	0.9129	0.6164	0.4986	0.5348	0.7460	0.5420
Qx_Limb_Muscle	**0.9941**	0.9331	0.8118	0.4521	0.9875	0.7341	0.6999	0.7682	0.7308	0.7661	0.9311	0.4484
Qx_Spleen	0.9221	**0.9252**	0.6012	0.1420	0.9005	0.6767	0.7891	0.4229	0.4438	0.4693	0.8984	0.0675
Qx_Trachea	0.5546	**0.9052**	0.6288	0.1248	0.5069	0.4966	0.4834	0.2689	0.4720	0.5154	0.5476	0.1008
Romanov	**0.8059**	0.7612	0.5662	0.3562	0.6715	0.3415	0.3959	0.3548	0.4899	0.5262	0.5778	0.2341
Tosches_turtle	0.7383	0.6133	0.5547	**0.7542**	0.3907	0.4825	0.5196	0.4847	0.7183	0.7088	0.3819	0.4565
Wang_Lung	0.7323	0.9077	0.8139	0.2044	0.9462	0.2627	0.9233	0.8716	0.3542	0.3391	**0.9745**	0.8607
Young	0.6996	0.6946	0.3474	0.6607	0.6292	0.4571	0.5960	0.3770	0.6286	**0.7089**	0.6562	0.2590
Average	**0.8223**	0.7909	0.6111	0.4988	0.7477	0.5182	0.5255	0.4454	0.3534	0.4108	0.7087	0.2887

Supplementary Table S16: Performance comparison of the different clustering algorithms on twenty real scRNA-seq datasets measured by ASW

Algorithm	scBGEDA	scziDesk	SHARP	DESC	scDeepCluster	CIDR	SOUP	SC3	Scanpy	Seurat	DCA	PCA
Adam	**0.6298**	0.6111	-0.0684	0.5865	0.5736	-0.0675	0.3419	0.3612	0.3651	0.5466	0.5741	-0.1619
Bach	0.5661	0.6193	-0.0348	0.3341	0.5156	0.5399	**0.6667**	0.3807	0.3669	0.4568	0.5833	0.3352
Chen	**0.5897**	0.5091	0.1043	0.3347	0.4330	-0.2303	0.3059	0.3046	0.3081	0.4945	0.5041	0.2876
Klein	0.7265	0.5901	0.1216	0.6684	0.6347	0.1752	0.6775	0.6919	0.6965	**0.8269**	0.6842	0.5999
Muraro	0.5933	0.5202	-0.2179	0.5156	0.4924	0.2555	0.4233	0.5475	0.5568	0.3324	**0.7564**	0.5162
Plasschaert	0.4536	0.4224	0.3626	0.2295	0.3690	0.3118	0.2413	0.4052	0.3338	**0.4813**	0.4688	0.3262
Pollen	**0.7783**	0.7362	-0.1618	0.7780	0.7674	0.4106	0.6346	0.7233	0.7289	0.6066	0.7280	0.4390
QS_Diaphragm	**0.8772**	0.8487	0.4568	0.8484	0.7758	0.7946	0.6320	0.6942	0.7092	0.6797	0.8366	0.0703
QS_Heart	0.7176	0.7129	0.5036	0.5334	0.6467	0.7347	0.6772	0.6214	0.6320	0.6603	**0.7618**	0.2404
QS_Limb_Muscle	**0.8524**	0.8360	0.5720	0.8486	0.7638	0.8008	0.7445	0.7097	0.7192	0.7233	0.8201	-0.0096
QS_Lung	**0.6289**	0.5448	0.2627	0.3304	0.4725	0.5851	0.4716	0.5537	0.5462	0.5241	0.5525	-0.0343
QS_Trachea	**0.6655**	0.5550	0.3754	0.3395	0.5669	0.6188	0.4700	0.6155	0.6146	0.5725	0.6038	0.1251
Qx_Bladder	0.7609	**0.7856**	0.4074	0.5072	0.6674	0.7408	0.7816	0.6839	0.6862	0.4837	0.6884	0.4533
Qx_Limb_Muscle	0.8262	**0.8274**	0.6767	0.6669	0.7866	0.6554	0.6675	0.7557	0.7680	0.7964	0.8223	0.6086
Qx_Spleen	0.6772	**0.7273**	0.2150	0.1505	0.6116	0.4294	0.6168	0.4535	0.4553	0.4188	0.7096	0.1466
Qx_Trachea	0.5018	**0.5967**	0.2177	0.1257	0.4464	0.2723	0.4853	0.5308	0.5324	0.5065	0.5101	0.2462
Romanov	**0.4517**	0.3831	0.3129	0.2455	0.3462	-0.0247	0.2073	0.3481	0.3434	0.3150	0.3499	0.2198
Tosches_turtle	**0.5998**	0.4852	0.0697	0.4110	0.3943	0.3663	0.4583	0.3496	0.3391	0.4584	0.4439	0.3151
Wang_Lung	0.4380	0.6947	**0.7733**	0.3532	0.6232	0.0910	0.6353	0.4302	0.4146	0.3689	0.5846	0.4421
Young	0.4526	0.4159	-0.1183	**0.5351**	0.4217	0.1336	0.1354	0.1478	0.1339	0.2858	0.4145	-0.0013
Average	**0.6394**	0.6211	0.2415	0.4671	0.5654	0.3797	0.5137	0.5154	0.5125	0.5269	0.6198	0.2582

Supplementary Table S17: Performance comparison of the different clustering algorithms on twenty real scRNA-seq datasets measured by cLISI

Algorithm	scBGEDA	scziDesk	SHARP	DESC	scDeepCluster	CIDR	SOUP	SC3	Scanpy	Seurat	DCA	PCA
Adam	**1.0000**	0.8967	0.9021	0.9895	0.9457	0.5581	0.8599	0.7636	0.7659	0.9174	0.8617	0.4935
Bach	**1.0000**	0.9943	0.9149	**1.0000**	0.9916	1.0000	1.0000	0.9918	0.9914	0.9992	0.9999	0.9358
Chen	**1.0000**	0.9802	0.9773	0.9988	0.9902	0.9728	0.9686	0.9560	0.9556	0.9985	0.9942	0.9550
Klein	**1.0000**	0.9919	0.9171	**1.0000**	0.9960	0.9878	0.9993	0.9981	0.9981	**1.0000**	0.9894	0.9355
Muraro	**1.0000**	0.8706	0.8800	0.9986	0.9806	0.9314	0.9931	0.8552	0.8566	0.9897	0.9484	0.9932
Plasschaert	**1.0000**	0.9982	0.9920	**1.0000**	1.0000	0.9987	0.9997	0.9953	0.9954	**1.0000**	**1.0000**	0.9622
Pollen	**1.0000**	0.9065	0.9045	0.9868	0.9268	0.9361	0.8732	0.8772	0.8852	0.9218	0.8691	0.8911
QS_Diaphragm	**1.0000**	0.9938	1.0000	**1.0000**	**1.0000**	1.0000	0.9997	**1.0000**	**1.0000**	1.0000	0.9990	0.8134
QS_Heart	**1.0000**	0.9881	0.9988	**1.0000**	**1.0000**	**1.0000**	1.0000	0.9964	0.9961	**1.0000**	**1.0000**	0.9320
QS_Limb_Muscle	**1.0000**	0.9970	1.0000	**1.0000**	**1.0000**	0.9998	**1.0000**	0.9939	0.9943	1.0000	0.9996	0.8502
QS_Lung	**1.0000**	0.9842	0.9884	0.9997	0.9960	0.9987	0.9965	0.9372	0.9377	0.9976	0.9858	0.8633
QS_Trachea	**1.0000**	0.9853	0.9981	**1.0000**	0.9899	1.0000	0.9968	0.9980	0.9980	0.9993	0.9799	0.7395
Qx_Bladder	**1.0000**	**1.0000**	0.9554	**1.0000**	**1.0000**	**1.0000**	**1.0000**	**1.0000**	**1.0000**	**1.0000**	**1.0000**	0.9881
Qx_Limb_Muscle	**1.0000**	0.9937	0.9769	**1.0000**	**1.0000**	0.9918	0.9995	0.9568	0.9580	1.0000	0.9992	0.9627
Qx_Spleen	**1.0000**	**1.0000**	0.9942	**1.0000**	**1.0000**	1.0000	**1.0000**	0.9987	0.9987	**1.0000**	**1.0000**	0.9536
Qx_Trachea	**1.0000**	0.9991	0.9992	**1.0000**	**1.0000**	0.9966	0.9959	0.9946	0.9945	**1.0000**	**1.0000**	0.8691
Romanov	**1.0000**	0.8877	0.9432	0.9848	0.9371	0.9360	0.9071	0.9171	0.9161	0.9824	0.8788	0.8825
Tosches_turtle	**1.0000**	0.9300	0.9546	0.9992	0.9538	0.9301	0.9577	0.9362	0.9371	0.9652	0.9567	0.9196
Wang_Lung	**1.0000**	**1.0000**	1.0000	**1.0000**	**1.0000**	1.0000	**1.0000**	**1.0000**	**1.0000**	**1.0000**	**1.0000**	**1.0000**
Young	**1.0000**	0.9196	0.9759	0.9924	0.9265	0.8980	0.9081	0.8964	0.8961	0.9148	0.9058	0.8506
Average	**1.0000**	0.9658	0.9636	0.9975	0.9817	0.9568	0.9728	0.9531	0.9537	0.9843	0.9684	0.8895

Supplementary Table S18: Wilcoxon test analysis of significant difference between the different clustering algorithms and scBGEDA measured by NMI on twenty scRNA-seq datasets

Dataset	scziDesk	SHARP	DESC	scDeepCluster	CIDR	SOUP	SC3	Scanpy	Seurat	DCA	PCA
Adam	0.0008	0.0002	0.0002	0.0002	0.0001	0.0002	0.0002	0.0001	0.0001	0.0002	0.0002
Bach	0.0002	0.0002	0.0002	0.0036	0.0001	0.0002	0.0002	0.0002	0.0001	0.0003	0.0002
Chen	0.0002	0.7337	0.0002	0.0002	0.0001	0.0002	0.0002	0.0001	0.0001	0.0002	0.0002
Klein	0.0010	0.0002	0.0008	0.0376	0.0001	0.0001	0.0002	0.0002	0.0001	0.0002	0.0001
Muraro	0.0002	0.0002	0.0002	0.0002	0.0001	0.0002	0.0002	0.0001	0.0001	0.0257	0.0002
Plasschaert	0.0003	0.0002	0.0211	0.0257	0.1153	0.0028	0.0640	0.0054	0.4429	0.0257	0.0002
Pollen	0.0022	0.0002	0.9096	0.0452	0.0172	0.0002	0.3075	0.1153	0.0001	0.4725	0.7909
QS_Diaphragm	0.0002	0.0002	0.0004	0.0017	0.0014	0.0001	0.0002	0.0002	0.0001	0.0090	0.0001
QS_Heart	0.0002	0.0002	0.0002	0.0003	0.0001	0.0002	0.0002	0.0002	0.0001	0.0002	0.0002
QS_Limb_Muscle	0.0002	0.0002	0.0002	0.0022	0.0001	0.0001	0.0002	0.0002	0.0001	0.0002	0.0001
QS_Lung	0.3847	0.3445	0.0022	0.0073	0.0001	0.0001	0.0002	0.0001	0.0001	0.1212	0.0002
QS_Trachea	0.0002	0.0002	0.0002	0.0003	0.0001	0.0001	0.0002	0.0001	0.0001	0.0002	0.0002
Qx_Bladder	0.0003	0.0002	0.0002	0.0002	0.0001	0.0001	0.0002	0.0001	0.0001	0.0002	0.0002
Qx_Limb_Muscle	0.0002	0.0002	0.0002	0.0002	0.0001	0.0001	0.0002	0.0001	0.0001	0.0002	0.0002
Qx_Spleen	0.0173	0.0002	0.0002	0.0452	0.0001	0.0001	0.0002	0.0002	0.0001	0.0002	0.0002
Qx_Trachea	0.0028	0.4274	0.0002	0.3447	0.0001	0.0001	0.0002	0.0002	0.0001	0.0028	0.0002
Romanov	0.0452	0.0002	0.0002	0.0004	0.0001	0.0001	0.0002	0.0001	0.0001	0.0002	0.0002
Tosches_turtle	0.0173	0.0002	0.0002	0.0002	0.0001	0.0002	0.0002	0.0311	0.0001	0.0002	0.0002
Wang_Lung	0.0002	0.0002	0.0002	0.0002	0.0001	0.0001	0.1405	0.0002	0.0001	0.0002	0.0001
Young	0.3447	0.0002	0.1405	0.0002	0.0001	0.0002	0.0002	0.0001	0.0001	0.0211	0.0002

Supplementary Table S19: Wilcoxon test analysis of significant difference between the different clustering algorithms and scBGEDA measured by ARI on twenty scRNA-seq datasets

Dataset	scziDesk	SHARP	DESC	scDeepCluster	CIDR	SOUP	SC3	Scanpy	Seurat	DCA	PCA
Adam	0.0003	0.0002	0.0002	0.0002	0.0001	0.0002	0.0002	0.0001	0.0001	0.0002	0.0002
Bach	0.0002	0.0002	0.0002	0.0002	0.0001	0.0002	0.0002	0.0002	0.0001	0.0002	0.0002
Chen	0.0002	0.0002	0.0002	0.0002	0.0001	0.0002	0.0002	0.0001	0.0001	0.0640	0.0006
Klein	0.0257	0.0002	0.0036	0.0757	0.0001	0.0001	0.0002	0.0002	0.0001	0.0012	0.0001
Muraro	0.0002	0.0002	0.0002	0.0002	0.0001	0.0002	0.0002	0.0001	0.0001	0.0017	0.0002
Plasschaert	0.0028	0.0028	0.0008	0.0002	0.0001	0.0028	0.0002	0.0002	0.0001	0.0002	0.0002
Pollen	0.4274	0.0002	0.1613	0.0008	0.0014	0.0002	0.7913	0.0014	0.0001	0.4272	0.0035
QS_Diaphragm	0.0002	0.0002	0.0004	0.0002	0.0014	0.0001	0.0002	0.0002	0.0001	0.0028	0.0001
QS_Heart	0.0002	0.0002	0.0002	0.0002	0.0001	0.0002	0.0002	0.0002	0.0001	0.0002	0.0002
QS_Limb_Muscle	0.0002	0.0002	0.0002	0.0010	0.0001	0.0001	0.0002	0.0002	0.0001	0.0002	0.0001
QS_Lung	0.0046	0.2121	0.0002	0.0006	0.0001	0.0001	0.0002	0.0001	0.0001	0.0004	0.0002
QS_Trachea	0.0002	0.0006	0.0002	0.0002	0.0001	0.0001	0.0002	0.0001	0.0001	0.0002	0.0002
Qx_Bladder	0.0002	0.0002	0.0002	0.0002	0.0001	0.0001	0.0002	0.0001	0.0001	0.0002	0.0002
Qx_Limb_Muscle	0.0002	0.0002	0.0002	0.0002	0.0001	0.0001	0.0002	0.0001	0.0001	0.0002	0.0002
Qx_Spleen	0.7337	0.0002	0.0002	0.0058	0.0001	0.0001	0.0002	0.0002	0.0001	0.0002	0.0002
Qx_Trachea	0.0028	0.0002	0.0002	0.0003	0.0001	0.0001	0.0002	0.0002	0.0001	0.2123	0.0002
Romanov	0.0002	0.0002	0.0002	0.0002	0.0001	0.0001	0.0002	0.0001	0.0001	0.0002	0.0002
Tosches_turtle	0.0173	0.0028	0.1859	0.0002	0.0001	0.0002	0.0002	0.0002	0.0001	0.0002	0.0002
Wang_Lung	0.0002	0.0028	0.0002	0.0002	0.0001	0.0001	0.1405	0.0002	0.0001	0.0002	0.0001
Young	0.9698	0.0002	0.0002	0.0002	0.0001	0.0002	0.0002	0.0001	0.0001	0.0002	0.0002

Supplementary Table S20: Clustering result comparison of scBGEDA with or without MSE loss measured by ARI

DataSet	scBGEDA	scBGEDA without MSE loss
Adam	0.9061	**0.9209**
Bach	**0.8453**	0.8397
Chen	0.3590	**0.3749**
Klein	**0.8688**	0.8192
Muraro	0.9271	**0.9293**
Plasschaert	0.5957	**0.6650**
Pollen	**0.8759**	0.8740
QS_Diaphragm	**0.9830**	0.9820
QS_Heart	**0.9647**	0.9641
QS_Limb_Muscle	0.9801	**0.9832**
QS_Lung	**0.7660**	0.7568
QS_Trachea	**0.9309**	0.9296
Qx_Bladder	**0.9960**	0.9945
Qx_Limb_Muscle	0.9941	**0.9941**
Qx_Spleen	**0.9221**	0.9210
Qx_Trachea	0.5546	**0.5592**
Romanov	**0.8059**	0.7952
Tosches_turtle	0.7383	**0.7442**
Wang_Lung	0.7323	**0.7343**
Young	**0.6996**	0.6949

Supplementary Table S21: Wilcoxon test analysis of different algorithms with scBGEDA (p-value) on PBMC_68k

Wilcoxon test	scziDesk	SHARP	DESC	scDeepCluster	SOUP	SC3	Scanpy	Seurat	DCA	PCA
scBGEDA (PBMC_68k)	1.63E-04	1.83E-04	1.83E-04	0.0028	6.39E-05	1.83E-04	6.39E-05	6.39E-05	1.83E-04	1.83E-04

Supplementary Table S22: Wilcoxon test analysis of scBGEDA (p-value) with different data sizes of PMBC_68k

Wilcoxon test	20%	40%	60%	80%
scBGEDA (PBMC_68k)	0.2934	0.2372	0.0569	0.1891

Supplementary Table S23: Comparative performance of scBGEDA with different clustering algorithms measured by ASW and cLISI

	ASW			cLISI		
Dataset	scBGEDA	scBGEDA$_{Leiden}$	scBGDDA$_{Louvain}$	scBGEDA	scBGEDA$_{Leiden}$	scBGDDA$_{Louvain}$
Adam	0.6298	0.6311	**0.6331**	**1.0000**	0.8866	0.8861
Bach	0.5661	0.5765	**0.5767**	**1.0000**	**1.0000**	**1.0000**
Chen	0.5897	**0.5941**	0.5874	**1.0000**	0.9934	0.9947
Klein	0.7265	0.7204	**0.7346**	**1.0000**	0.9990	0.9992
Muraro	0.5933	0.5953	**0.6279**	**1.0000**	0.9931	0.9874
Plasschaert	**0.4536**	0.4416	0.4462	**1.0000**	**1.0000**	**1.0000**
Pollen	0.7783	**0.7883**	0.7811	**1.0000**	0.9260	0.9773
QS_Diaphragm	0.8772	**0.8785**	0.8743	**1.0000**	0.9998	0.9994
QS_Heart	0.7176	**0.7317**	0.7173	**1.0000**	**1.0000**	**1.0000**
QS_Limb_Muscle	**0.8524**	0.8423	0.8340	**1.0000**	0.9998	0.9993
QS_Lung	**0.6289**	0.6009	0.6146	**1.0000**	0.9953	0.9991
QS_Trachea	**0.6655**	0.6573	0.6330	**1.0000**	0.9995	0.9985
Qx_Bladder	0.7609	0.7652	**0.7792**	**1.0000**	**1.0000**	**1.0000**
Qx_Limb_Muscle	0.8262	0.8194	**0.8307**	**1.0000**	1.0000	1.0000
Qx_Spleen	0.6772	**0.6798**	0.6655	**1.0000**	**1.0000**	**1.0000**
Qx_Trachea	0.5018	0.5029	**0.5069**	**1.0000**	**1.0000**	**1.0000**
Romanov	0.4517	0.4525	**0.4547**	**1.0000**	0.9323	0.9392
Tosches_turtle	0.5998	0.5975	**0.6112**	**1.0000**	0.9512	0.9521
Wang_Lung	0.4380	**0.4384**	0.4244	**1.0000**	**1.0000**	**1.0000**
Young	**0.4526**	0.4228	0.4214	**1.0000**	0.9319	0.9293
Average	**0.6394**	0.6368	0.6377	**1.0000**	0.9804	0.9831

Supplementary Table S24: Comparative performance of scBGEDA with different dimension reduction algorithms measured by ASW and cLISI

	ASW				cLISI			
Dataset	scBGEDA	scBGEDA$_{UMAP}$	scBGEDA$_{PCA}$	scBGEDA$_{FA}$	scBGEDA	scBGEDA$_{UMAP}$	scBGEDA$_{PCA}$	scBGEDA$_{FA}$
Adam	**0.6298**	0.3232	0.5595	0.5071	**1.0000**	0.9561	0.9105	0.8643
Bach	**0.5661**	0.1976	0.4667	0.4336	**1.0000**	0.9978	0.9988	0.9878
Chen	**0.5897**	0.0154	0.4176	0.4294	**1.0000**	0.9944	0.9970	0.9945
Klein	0.7265	0.3536	**0.8022**	0.5389	**1.0000**	0.9318	**1.0000**	0.9957
Muraro	0.5933	0.2916	**0.6388**	0.5780	**1.0000**	0.9959	0.9435	0.9711
Plasschaert	**0.4536**	0.0444	0.4217	0.3699	**1.0000**	0.8728	**1.0000**	0.9986
Pollen	0.7783	0.3475	**0.8000**	0.6663	**1.0000**	0.9008	0.8992	**1.0000**
QS_Diaphragm	**0.8772**	0.2375	0.6974	0.7111	**1.0000**	0.8897	0.9959	0.9965
QS_Heart	**0.7176**	0.2430	0.5871	0.5383	**1.0000**	0.9896	0.9992	0.9976
QS_Limb_Muscle	**0.8524**	0.1407	0.6567	0.7019	**1.0000**	0.9520	0.9960	0.9985
QS_Lung	**0.6289**	0.2828	0.5739	0.2667	**1.0000**	0.9851	0.9878	0.9871
QS_Trachea	**0.6655**	0.1426	0.5683	0.3674	**1.0000**	0.9517	0.9971	0.9838
Qx_Bladder	**0.7609**	0.3428	0.4518	0.3480	**1.0000**	0.9974	**1.0000**	**1.0000**
Qx_Limb_Muscle	**0.8262**	0.4677	0.7697	0.7383	**1.0000**	0.9968	0.9999	0.9947
Qx_Spleen	**0.6772**	0.1587	0.2110	0.5074	**1.0000**	0.9995	**1.0000**	0.8625
Qx_Trachea	**0.5018**	0.1829	0.4260	0.3309	**1.0000**	0.9899	**1.0000**	0.9974
Romanov	**0.4517**	0.2769	0.3606	0.2809	**1.0000**	0.9592	0.9724	0.9705
Tosches_turtle	**0.5998**	-0.1193	0.5267	0.3972	**1.0000**	0.9524	0.9402	0.9667
Wang_Lung	**0.4380**	0.1029	0.4163	0.4184	**1.0000**	0.9774	**1.0000**	**1.0000**
Young	0.4526	0.2446	0.3297	**0.4946**	**1.0000**	0.9886	0.9137	0.9593
Average	**0.6394**	0.2139	0.5341	0.4812	**1.0000**	0.9639	0.9776	0.9763

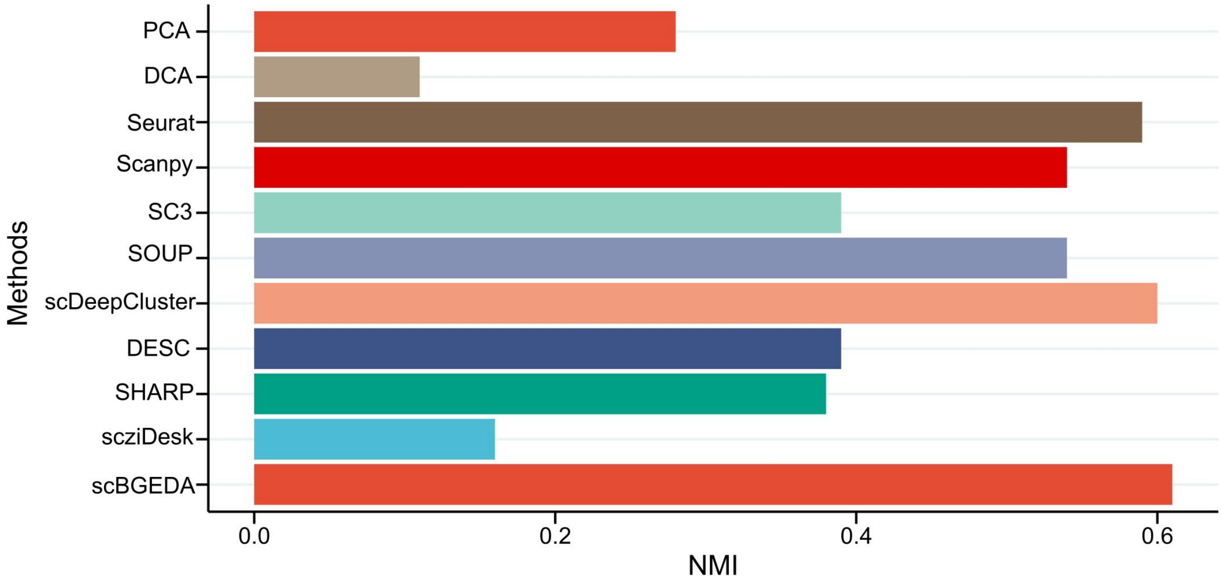

Supplementary Fig. S2: Results of comparative analysis of different clustering methods on the large-scale scRNA-seq dataset PBMC_68k measured by NMI.

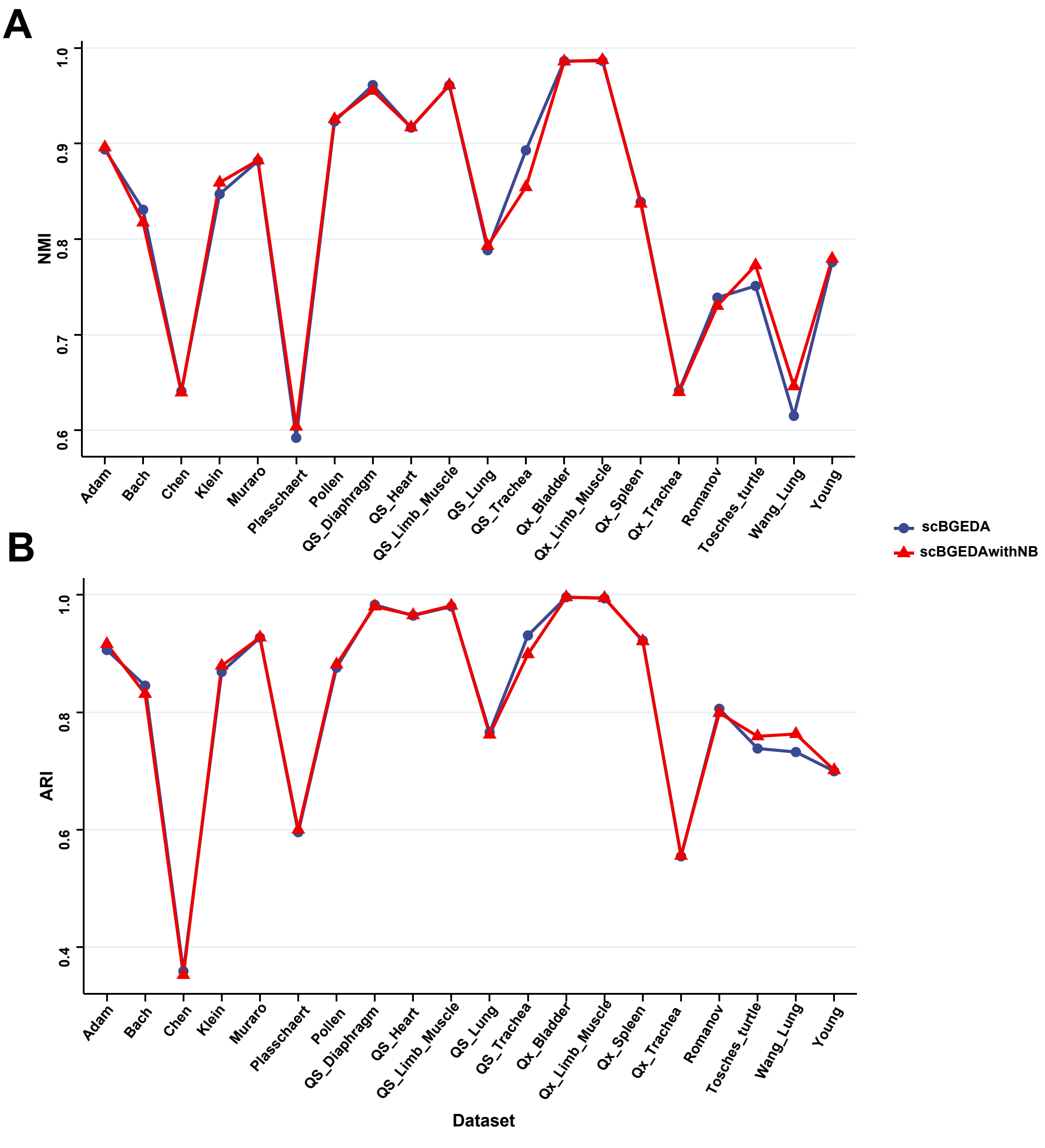

Supplementary Fig. S3: Comparative performance of scBGEDA and scBGEDA with NB model measured by NMI (A) and ARI (B).

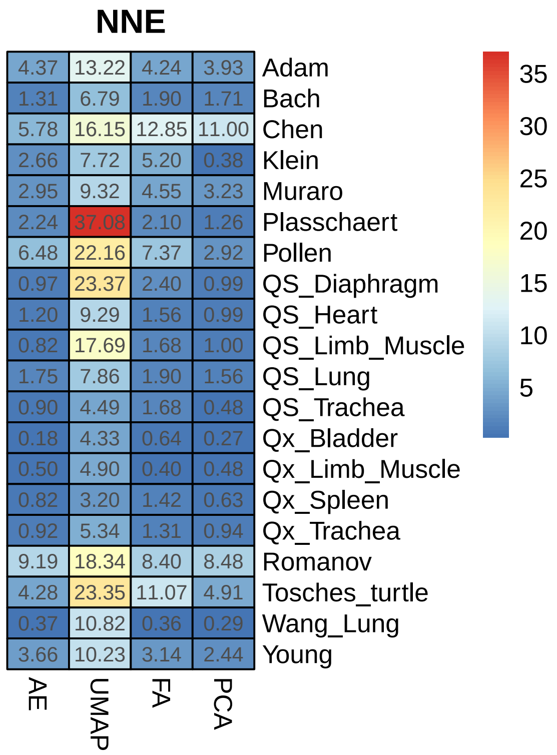

Supplementary Fig. S4: The nearest neighbor error values for different dimension reduction methods (in percent, lower is better), including the AE in scBGEDA, UMAP, FA, and PCA

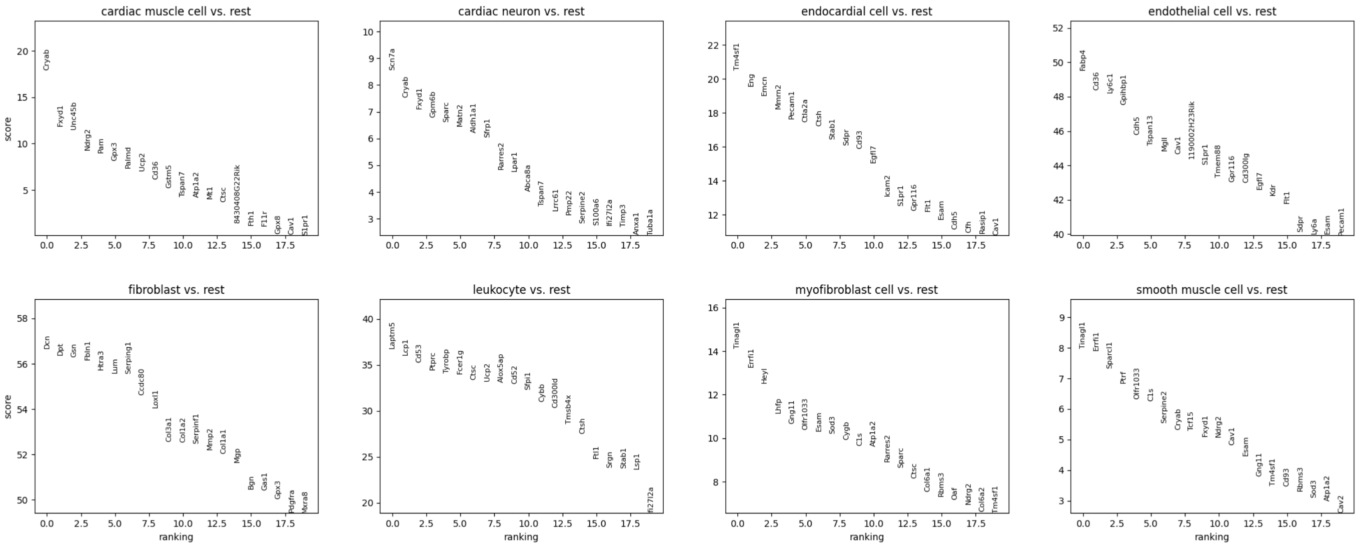

Supplementary Fig. S5: The gene ranking in different cell types by Wilcoxon rank-sum test.

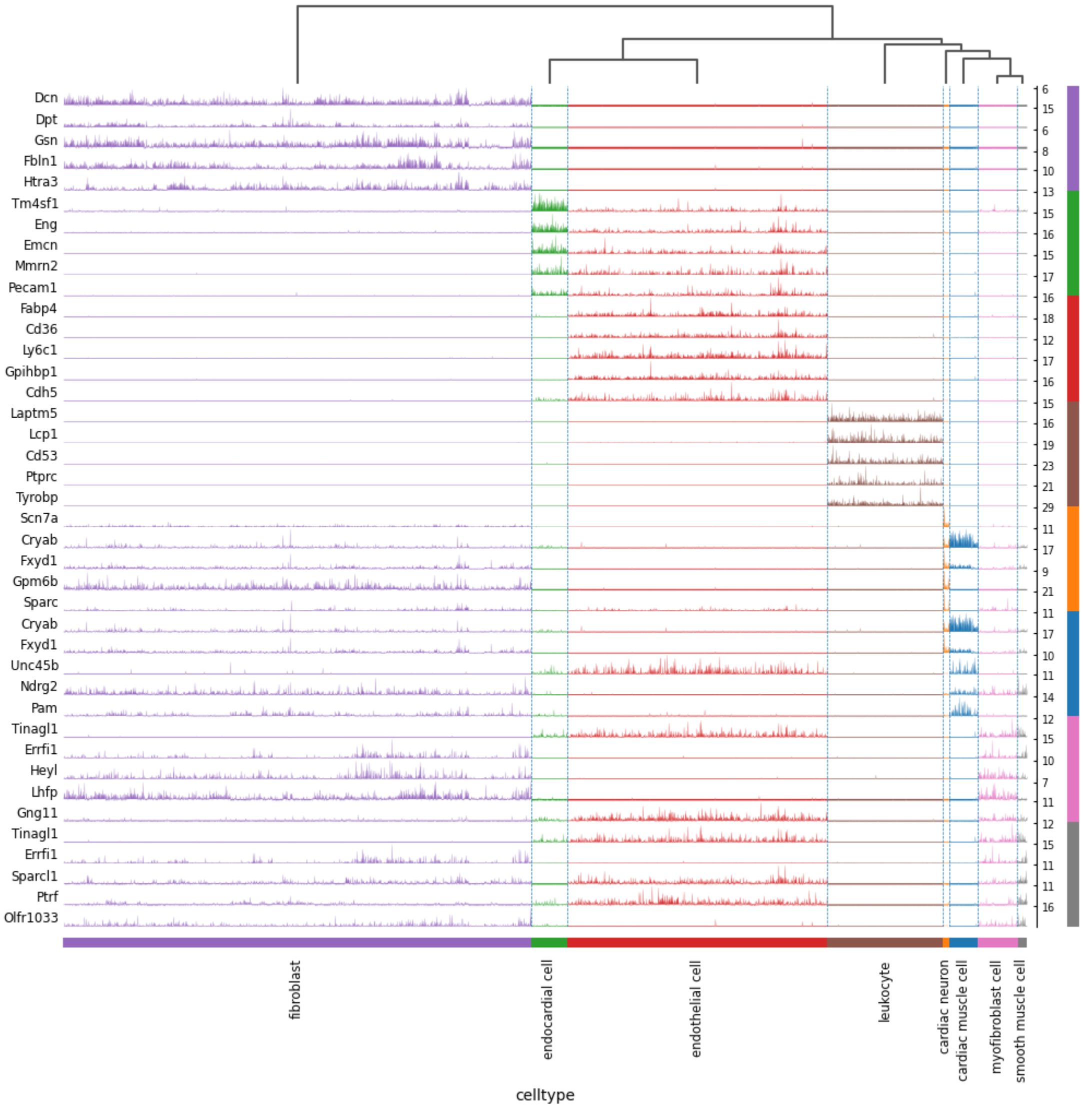

Supplementary Fig. S6: The track plot of top five differentially expressed genes (DEGs) within each cell type.

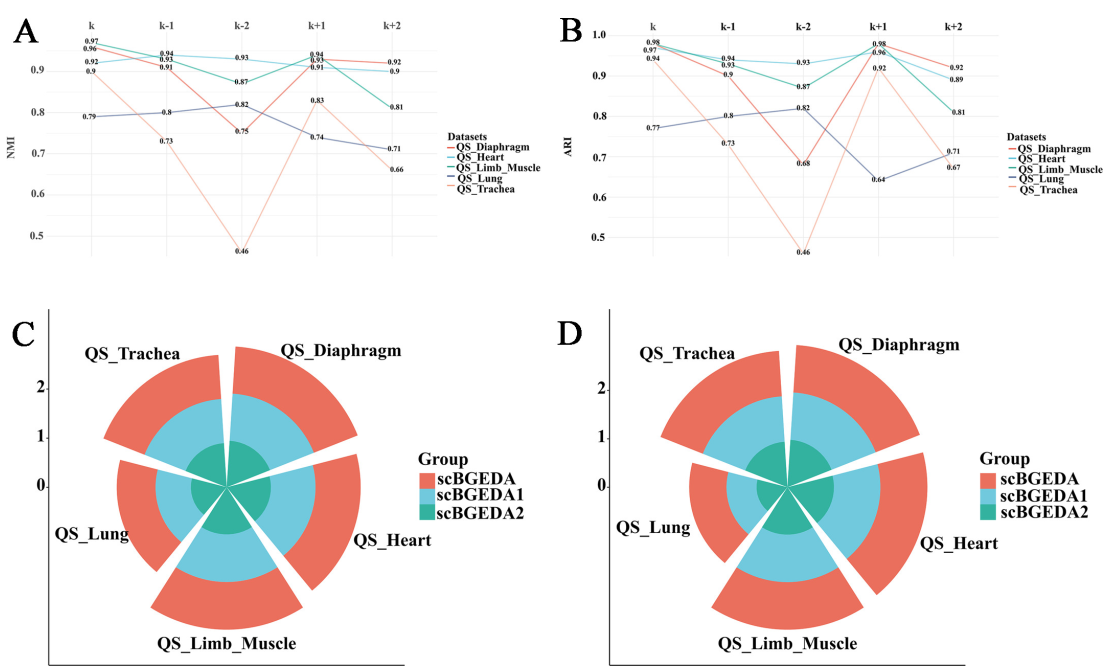

Supplementary Fig. S7: (A, B) Comparison NMI and ARI values of scBGEDA with different disturbed cluster numbers on five real scRNA-seq datasets from the Smart-seq2 platform. (C, D) Comparison NMI and ARI values of scBGEDA with different hidden layer numbers on five real scRNA-seq datasets.

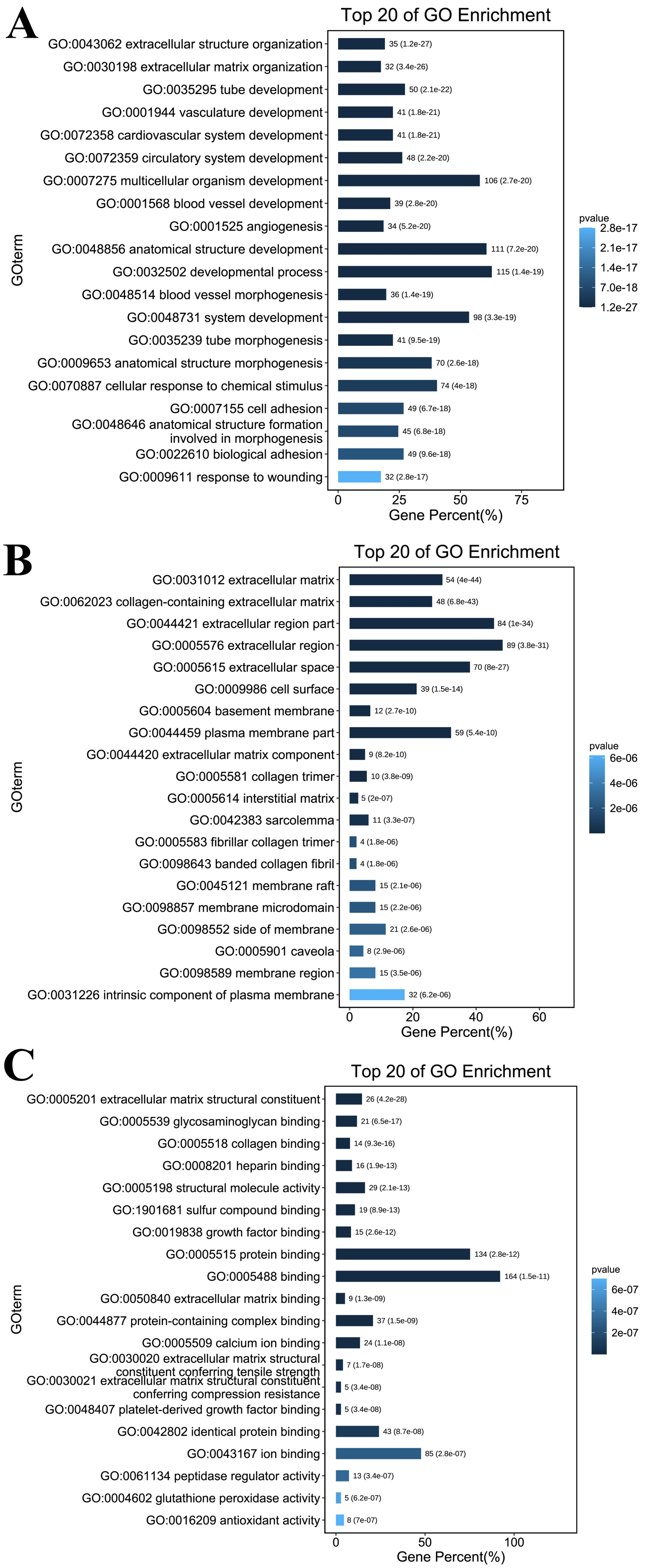

Supplementary Fig. S8: The top 20 GO categories ranked by p-value.

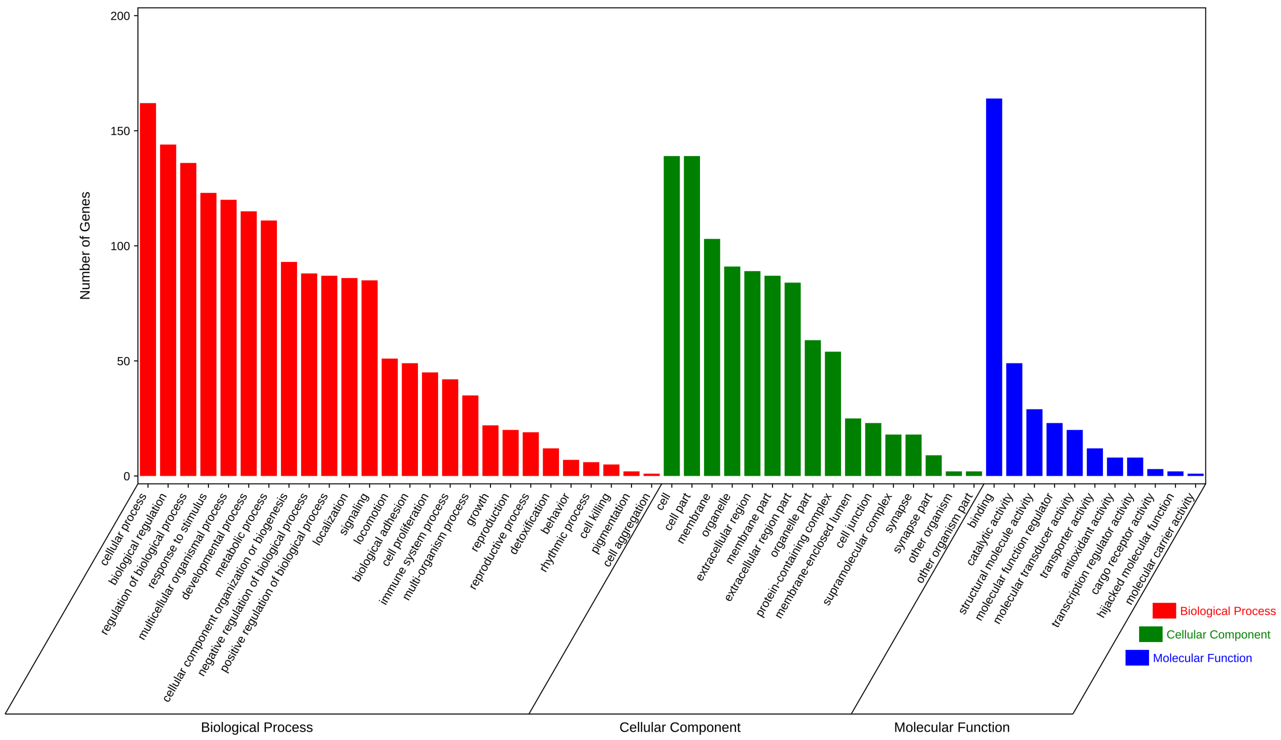

Supplementary Fig. S9: The gene-related GO distribution with three different types of GO enrichment.

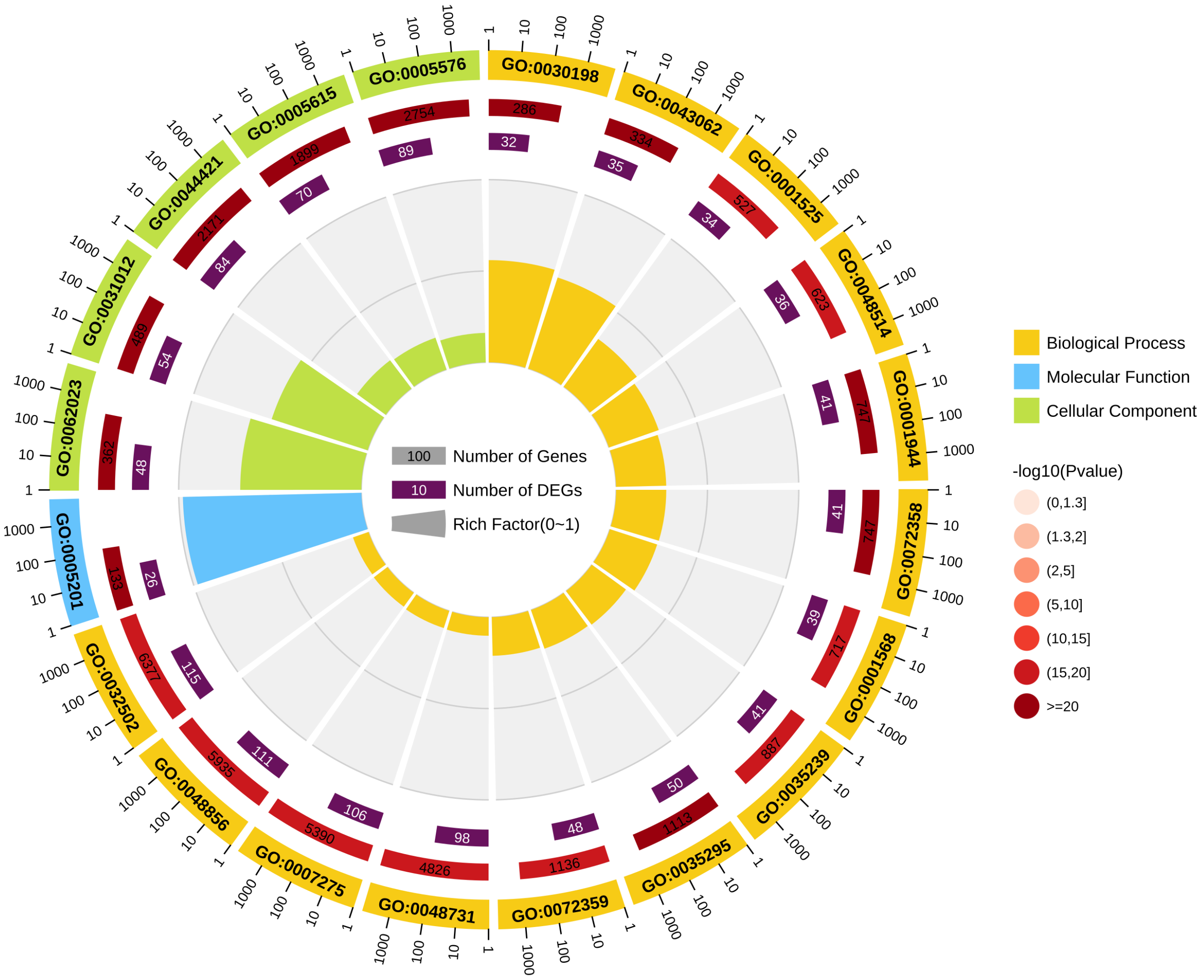

Supplementary Fig. S10: Circular visualization of the gene annotation enrichment analysis using biological processes, cellular components, and molecular functions components.

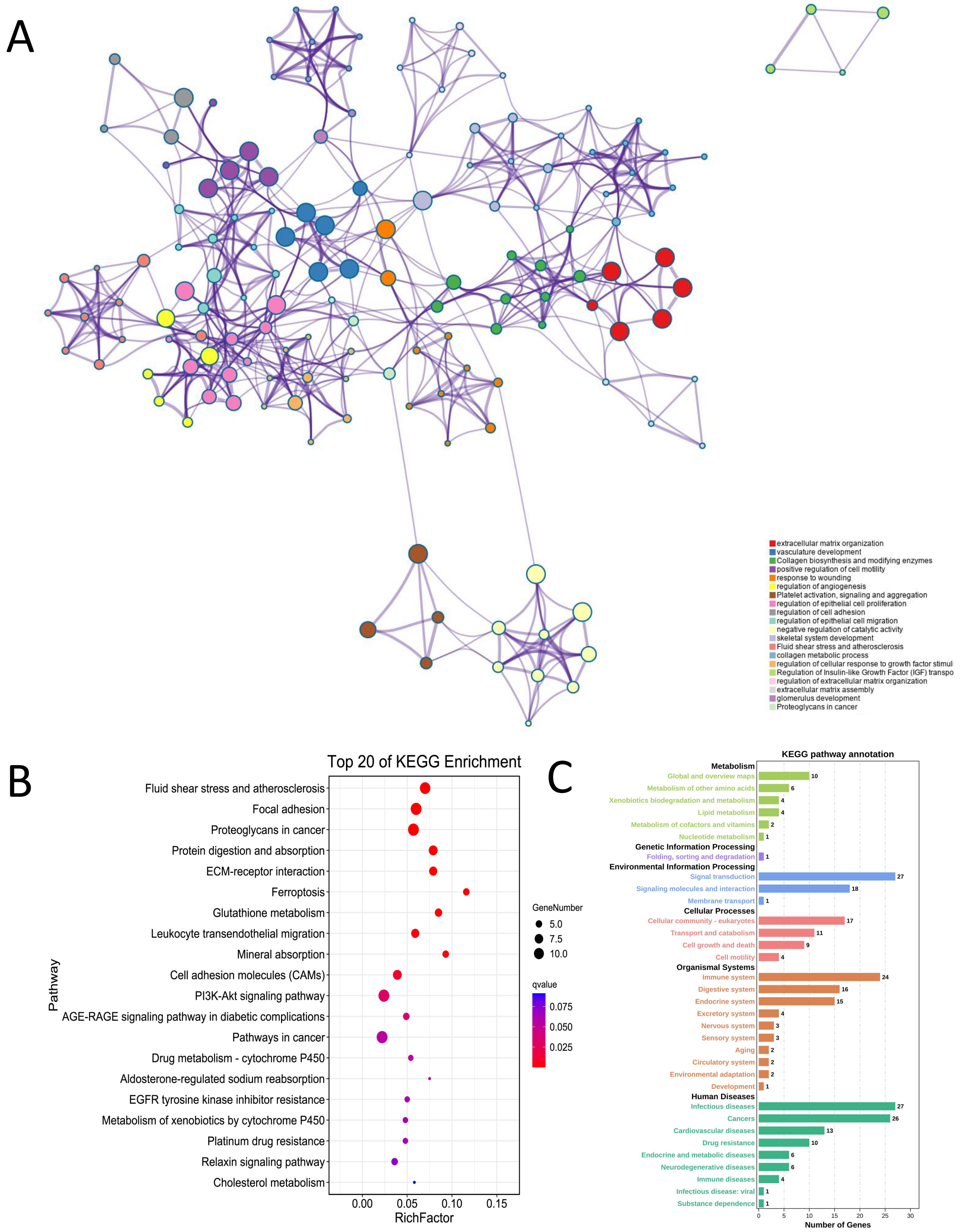

Supplementary Fig. S11: Genomic interpretability of scBGEDA. (A) The enrichment terms network is colored by the nodes representing the cluster ID, which are typically similar to each other when they pertain to the same cluster ID. (B) The top 20 KEGG enrichment terms ranked by p-values. (C) KEGG classification with various pathways including Metabolism, Genetic Information Processing, Environmental Information Processing, Cellular Processes, Organismal Systems, and Human Diseases.

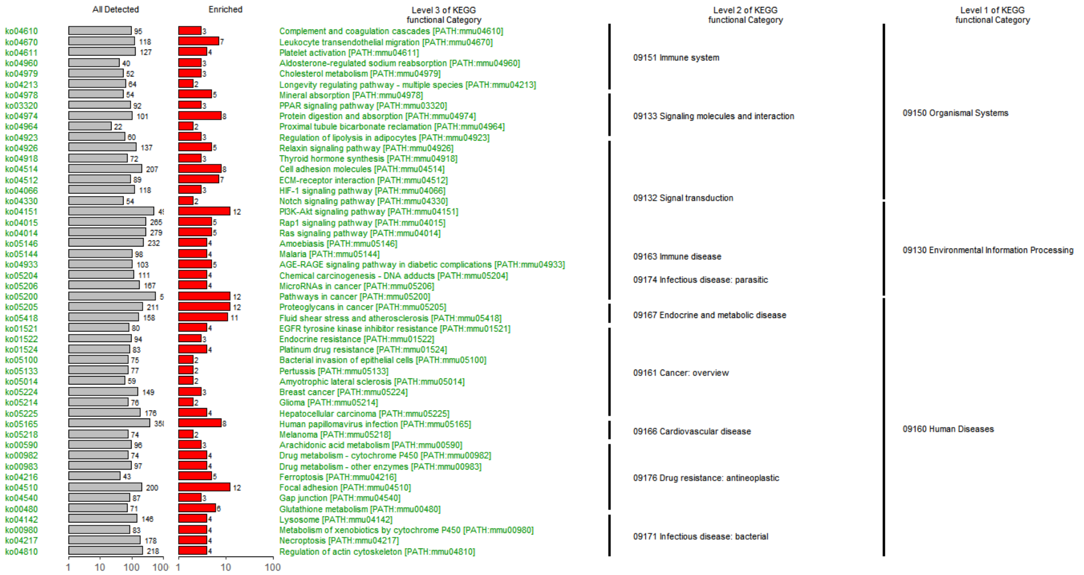

Supplementary Fig. S12: Three levels of KEGG functional categories and the number of enriched genes in the top 50 KEGG pathways ranked by p values.

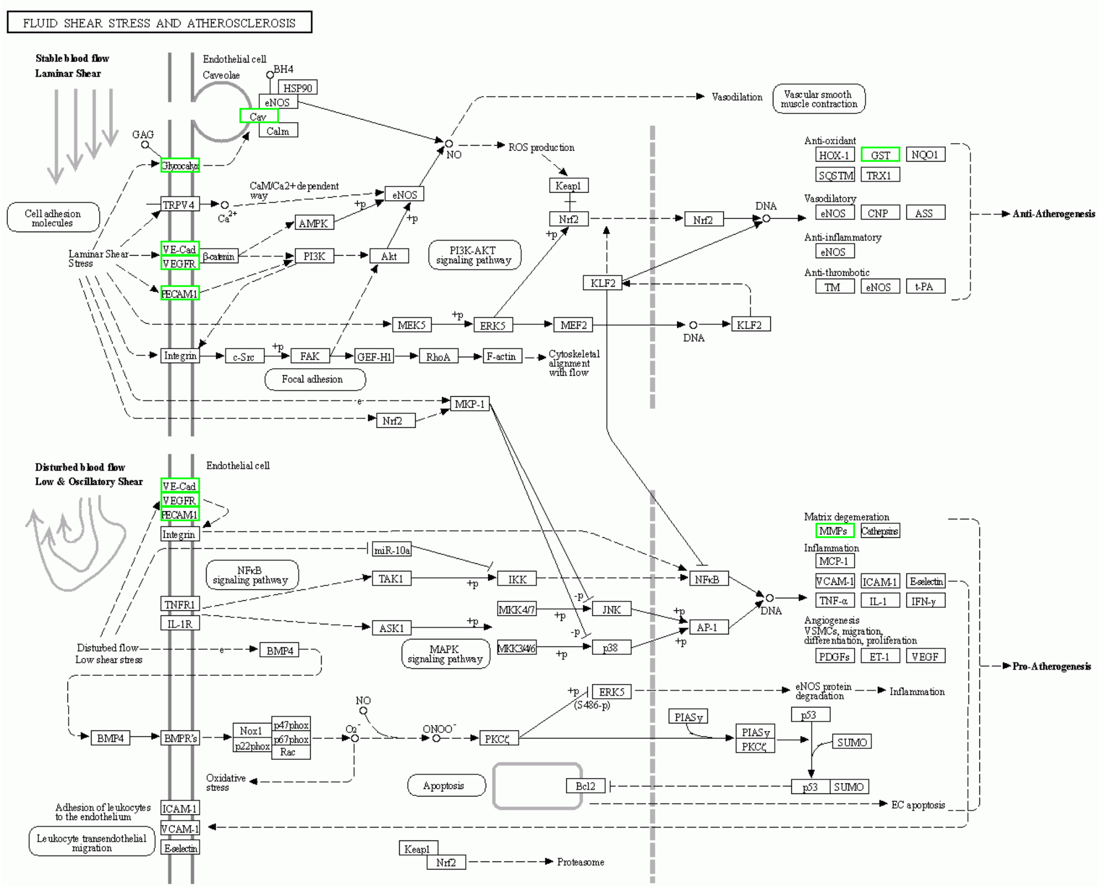

Supplementary Fig. S13: Fluid shear stress and atherosclerosis pathway (ko05418) provided by the KEGG database: those genes marked in green are the genes chosen by scBGEDA.

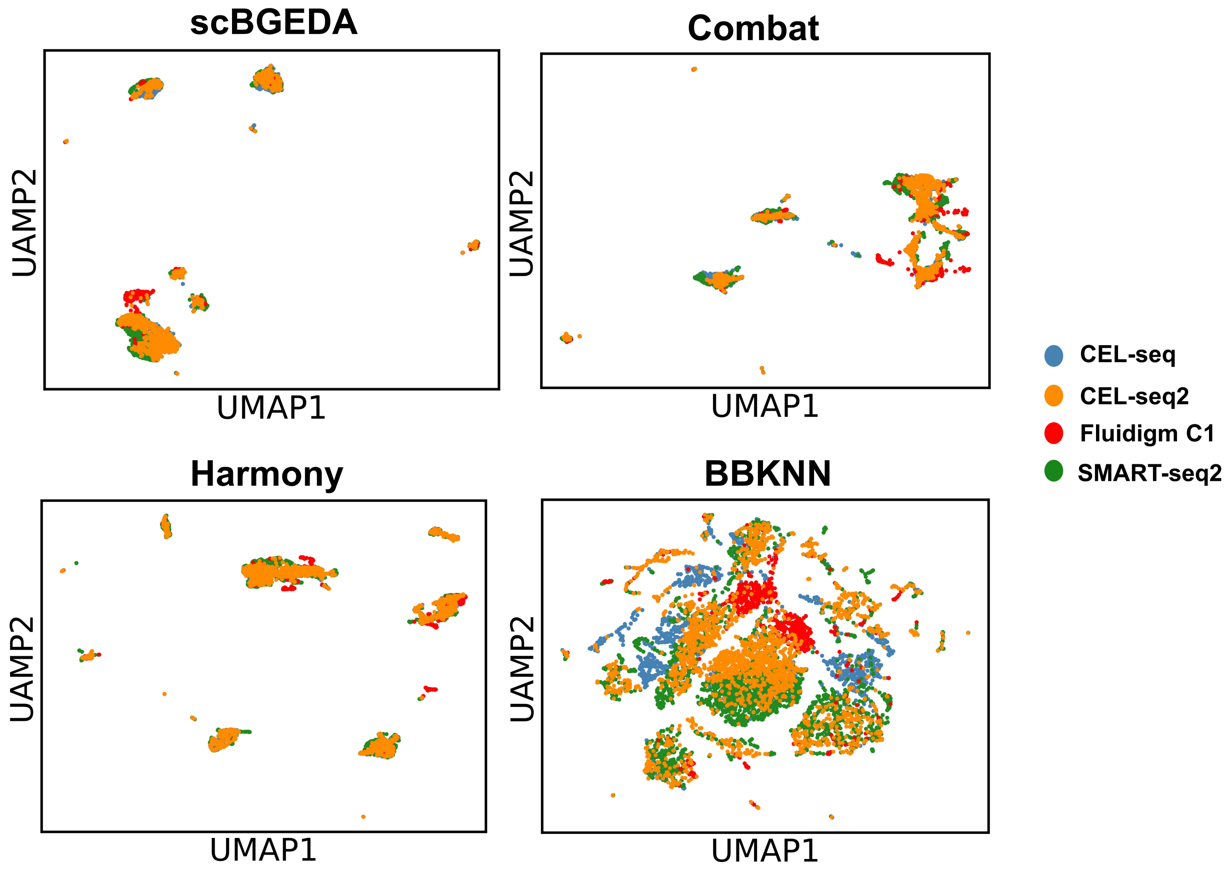

Supplementary Fig. S14: Comparison results of different batch effect correction methods on four human pancreas datasets by UMAP plots.

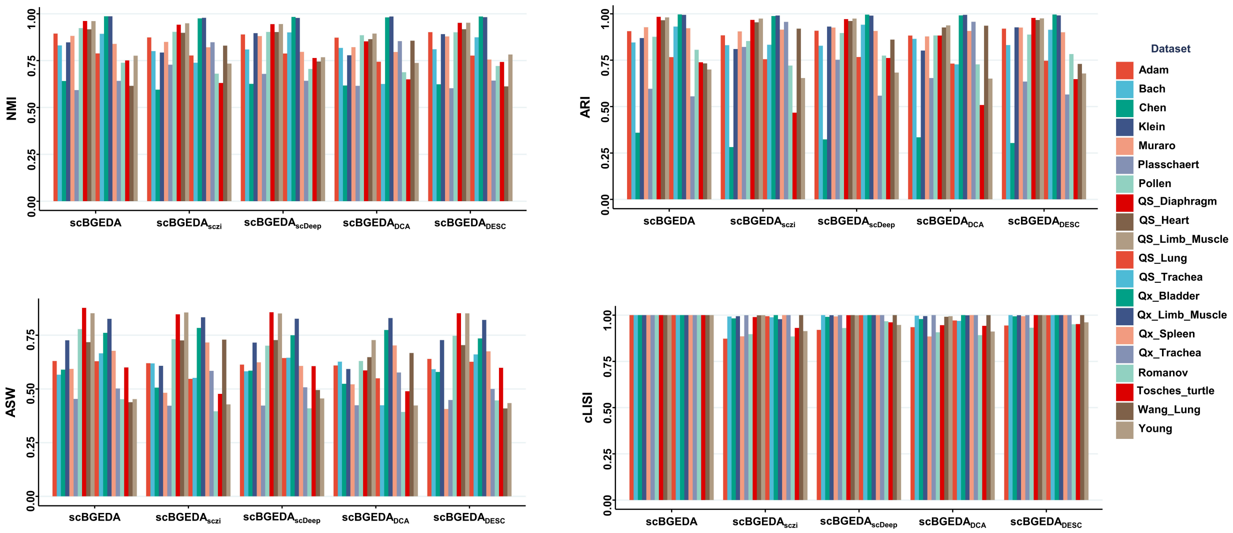

Supplementary Fig. S15: Comparative performance of scBGEDA with different default settings measured by NMI, ARI, ASW, and cLISI.
